# High Humidity Exacerbates Psoriasiform Skin Disease Relapse by Increasing Tissue‐Resident Memory T Cells via Altering Skin Microbiota

**DOI:** 10.1002/advs.202504061

**Published:** 2026-04-16

**Authors:** Chun‐Ling Liang, Yuchao Chen, Chuanjian Lu, Huazhen Liu, Fenglian Qin, Sisi Dai, Feifei Qiu, Haiming Chen, Weihui Lu, Jonathan S. Bromberg, Zhenhua Dai

**Affiliations:** ^1^ Section of Immunology Guangdong Provincial Academy of Chinese Medical Sciences Guangzhou Guangdong China; ^2^ Guangdong Provincial Hospital of Chinese Medicine Guangzhou Guangdong China; ^3^ Department of Microbiology and Surgery University of Maryland School of Medicine Baltimore Maryland USA

**Keywords:** humidity, keratinocyte, psoriasis, skin microbiome, tissue‐resident memory T cell (T_RM_)

## Abstract

Psoriasis recurrence remains a common and difficult medical problem, while environmental humidity appears to have an impact on psoriasis morbidity. However, it remains unknown whether and how high humidity impacts psoriasis relapse. Using unique mouse models of psoriasis relapse, we found that high humidity exposure exacerbated psoriasis recurrence by increasing skin‐resident memory CD8^+^ T (T_RM_) cells through a mechanism depending on its upregulation of IL‐15Rα on keratinocytes. Keratinocyte‐specific knockout of IL‐15Rα or administrating soluble sIL‐15Rα to block IL‐15 abrogated these effects. Moreover, the effects of high humidity on psoriasis relapse, IL‐15Rα expression and skin T_RM_ cell formation were attributed to cutaneous *Staphylococcus nepalensis* since its recolonization or its specific metabolite, asymmetric dimethylarginine (ADMA), upregulated IL‐15Rα expression on keratinocytes, increased skin CD8^+^ T_RM_ cells and worsened psoriasis relapse. However, treatment with mupirocin, an antibiotic, alleviated recurrent psoriasis. In vitro experiments showed that culture supernatant of *Staphylococcus nepalensis* upregulated IL‐15Rα expression on keratinocytes, while IL‐15Rα‐expressing keratinocytes promoted formation of CD8^+^ T_RM_ phenotypes. Finally, high humidity also aggravated psoriatic skin lesions in humanized mice. Thus, our findings enhanced a new understanding of how climatic factors govern psoriasis recurrence and unveiled a role for IL‐15Rα‐expressing keratinocytes in skin CD8^+^ T_RM_ formation and psoriasis relapse.

## Graphical abstract

We demonstrated that high humidity worsened psoriasis relapse in murine psoriasiform skin inflammation by increasing skin‐resident memory CD8^+^ cells via upregulating IL‐15Rα on keratinocytes. The increases in IL‐15Rα and memory CD8^+^ cells were attributed to *S. nepalensis* and its metabolite ADMA in skin exposed to high humidity. High humidity also promoted psoriasis recurrence in humanized mice engrafted with skin from psoriatic patients.

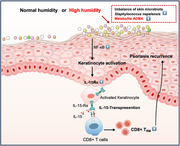

## 1 Introduction

Psoriasis is a chronic, recurrent, and autoinflammatory skin disease with a global prevalence of 0.1% to 11.43% [1]. The etiology of psoriasis is not fully understood. In addition to the complex interactions between the innate and adaptive immune system [2], psoriasis is characterized by seasonal and latitudinal variations. Climatic factors, including pollution, sunlight exposure, temperature, humidity, and seasonal changes, are correlated with the morbidity and aggravation of psoriasis [3, 4, 5]. Generally speaking, humid air may improve skin barrier function, while low humidity disrupts the skin epithelial barrier [6, 7] and promotes the epidermal IL‐1β secretion [8]. However, prolonged exposure to a high‐humidity environment may also lead to excessive skin hydration, weakened skin barrier, and increased susceptibility to external irritants [9]. Given the potential impacts of high humidity on the epithelial barrier, skin microbiota, and immune system, high humidity may alter the progression of psoriasis. Although humidity is somehow associated with morbidity rate of psoriasis [10, 11], it remains unknown whether and how high humidity impacts psoriasis relapse, a critical medical problem with psoriasis patients.

Recent studies have shown that the recurrence of psoriasis in the same place is associated with memory T cells, especially tissue‐resident memory T (T_RM_) cells [12, 13]. We previously demonstrated that in both normal murine models and humanized mouse models of psoriasis relapse [14], CD8^+^ T_RM_ cells were abundantly present in the skin and contributed to the relapse of psoriasis. Therefore, targeting memory T cells, particularly CD8^+^ T_RM_ cells, could be a promising strategy for preventing the recurrence of psoriasis. IL‐15 plays a crucial role in maintaining long‐term survival, function, and homeostatic expansion of T_RM_ cells [15, 16]. Local signaling by IL‐15 and TGFβ is sufficient to support T_RM_ development and survival in skin [17]. IL‐15 transduces signals through binding to its specific receptor, IL‐15Rα. In this process, IL‐15 forms a receptor complex with IL‐15Rα, which either directly transduces signaling in T cells expressing IL‐15α, IL‐2Rβ, and the common γ chain or indirectly presents IL‐15 to T cells. Indeed, IL‐15Rα on macrophages or DCs supports memory CD8^+^ T cell homeostasis by trans‐presenting IL‐15 [18]. Keratinocytes in the skin of psoriasis patients or mice also express IL‐15Rα [19], indicating that keratinocytes expressing IL‐15Rα may also play a role in cutaneous T_RM_ cell development.

The skin microbiome plays an essential role in maintaining skin homeostasis and health by regulating barrier function, inflammatory reactions, and immune responses [20, 21, 22], while microbial dysbiosis is associated with human psoriasis [23]. Mounting evidence has indicated the roles for microbiome in the pathogenesis, progression, and treatment of psoriasis [24, 25, 26, 27]. Moreover, cultural and environmental habits could affect the skin microbiome that in turn influences psoriasis [28]. Especially, skin microbiota modulates the local skin inflammation and resident T cell function [29]. On the other hand, high humidity has been shown to promote some bacterial growth [30]. However, it remains unclear how high humidity exactly alters skin microbiota, which in turn impacts psoriasis relapse.

In this study, we evaluated the effects of a high‐humidity environment on the relapse of psoriasis in imiquimod (IMQ)‐induced psoriatic mouse models. We found that high humidity exposure exacerbated the recurrence of psoriasis by promoting the accumulation of skin CD8^+^ T_RM_ cells through a mechanism that was dependent on IL‐15Rα on keratinocytes. Moreover, we demonstrated that the effects of high humidity on psoriasis relapse and T_RM_ cell formation were attributed to the enrichment of cutaneous *Staphylococcus nepalensis*, which in turn upregulated IL‐15Rα expression on keratinocytes. Finally, high humidity also aggravated human skin lesions in humanized mice that received skin grafts from psoriatic patients.

## 2 Results

### 2.1 High‐Humidity Exacerbates the Relapse of Psoriasiform Skin Inflammation

**FIGURE 1:**
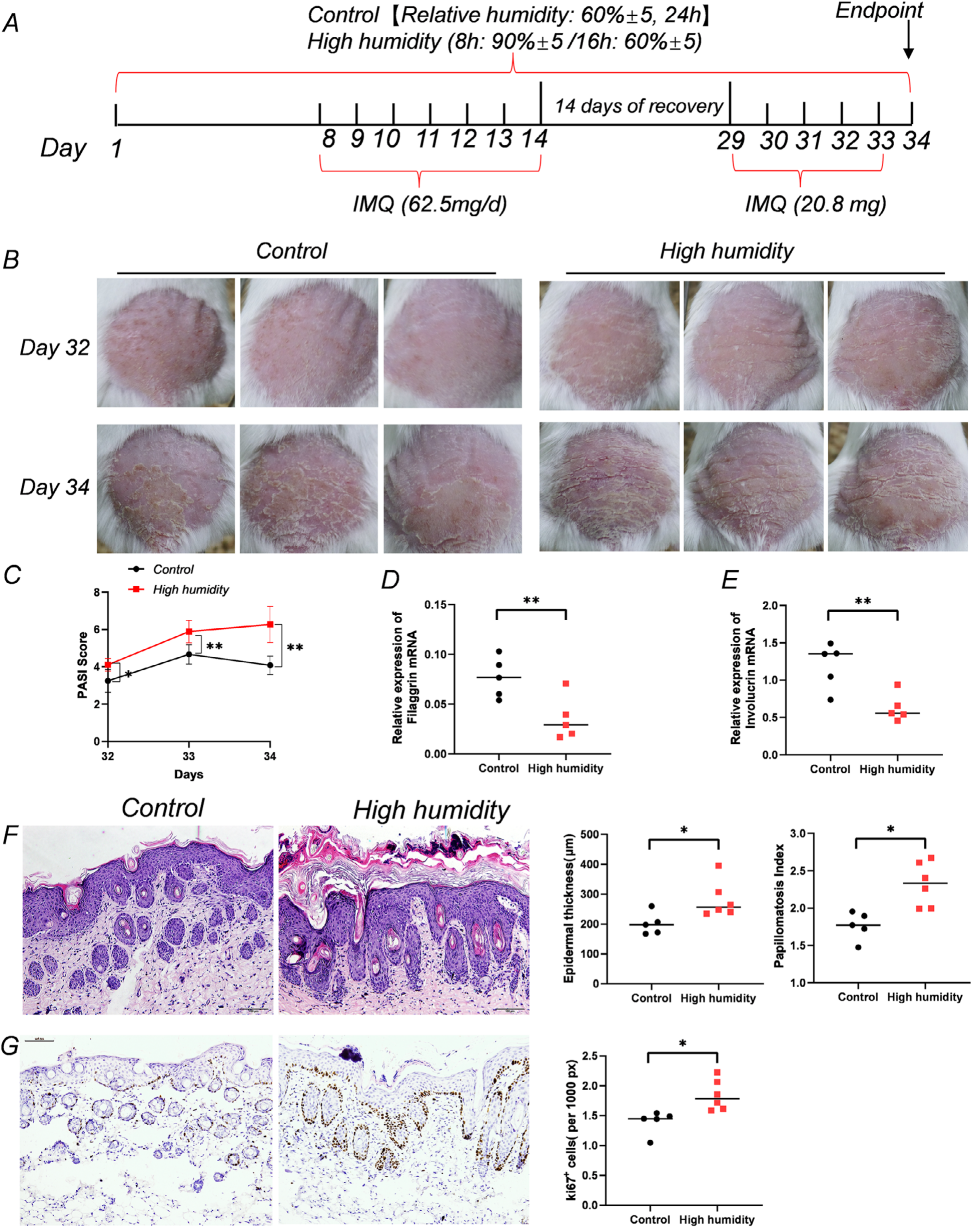
High humidity environment promotes the relapse of psoriasiform skin inflammation. BALB/c mice were kept in climate incubator at 25°C ± 2°C with a relative humidity of 90% ± 5% (High humidity group) or 60% ± 5% humidity (Control group) starting one week before the first round treatment of IMQ (62.5 mg). Following a 14‐day recovery period, all mice were rechallenged with a second round application of one‐third of the full dose (20.8 mg) for 5 days. (A) Experimental scheme; (B) Representative images of skin lesions during the relapse phase; (C) The PASI scores of skin lesions in psoriatic mice during the relapse phase; (D, E) The relative mRNA expressions of filaggrin and involucrin in the skin of psoriatic mice; (F) Images of H&E staining of skin sections, along with measurements of epidermal thickness and papillomatosis index (200×, Scale bar: 100 µm); (G) Representing pictures of Ki67 staining of skin sections and quantitation of Ki67^+^ cells in epidermis (200×, Scale bar: 100 µm). Data are presented as mean ± SD (n = 5–6 mice/group, ^*^ *p* *< *0.05 and ^**^ *p* *< *0.01). Shown is one representative from the three separate experiments.

Climate changes, such as temperature and humidity, may influence the morbidity and severity of psoriasis. To investigate the effects of high‐humidity environment on the psoriasis relapse, we established a mouse model of IMQ‐induced psoriasis relapse and an artificial climate box that simulates a high‐humidity environment (Normal humidity control: 60% ± 5% for 24 h and high humidity: 90% ± 5% for 8 h and 60% ± 5% for 16 h, Figure 1A). In this mouse model, the second round use of IMQ at much lower doses (20.8 mg) caused severe skin lesions, but otherwise would not do so without the first round IMQ at higher doses of 62.5 mg (Our preliminary observation). As depicted in Figure 1B, representative images showed that a high‐humidity environment worsened the skin lesions, as also confirmed by PASI scoring (Figure 1C). Furthermore, we determined the effects of high humidity on the skin barrier. As shown in Figure 1D,E, high humidity significantly lowered the mRNA expression of both filaggrin and involucrin. Subsequently, we assessed the epidermal hyperplasia through H&E staining and immunohistochemical analysis. As shown in Figure 1F, second IMQ stimulation with the lower doses induced obvious epidermal hyperplasia and hyperkeratosis. High humidity further increased the skin inflammation with an increase in epidermal thickness and papillomatosis index. Similarly, high humidity significantly enhanced Ki67^+^ expression in the epidermis compared to the control group (Figure 1G). Taken together, these results indicate that a high‐humidity environment aggravates the relapse of psoriatic skin lesions. Interestingly, high humidity did not alter the severity of primary psoriasiform skin lesions (data not shown).

### 2.2 High‐Humidity Increases Skin‐Resident Memory CD8^+^ T Cells in Mice With IMQ‐Induced Psoriasis Relapse

**FIGURE 2:**
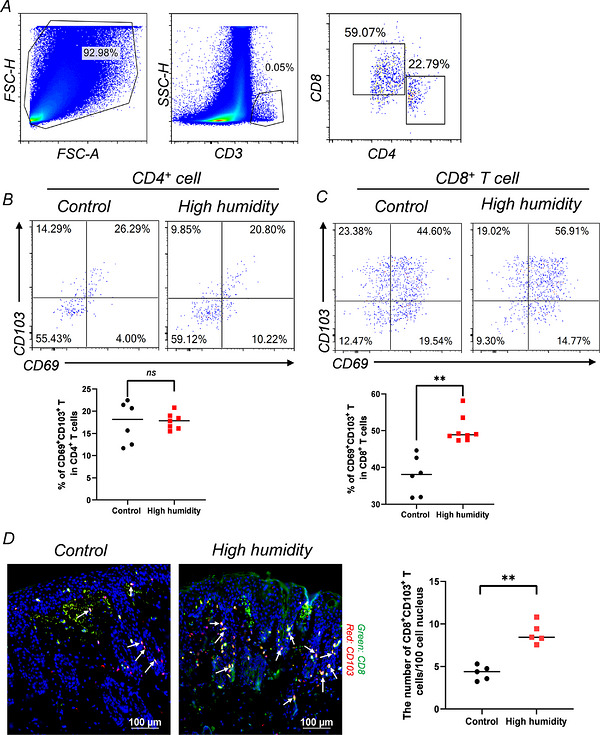
High humidity environment increases skin‐resident memory CD8^+^ T cells in IMQ‐induced mouse models of psoriasis relapse. Skin tissue of mice with psoriasis relapse was digested to prepare a single‐cell suspension for FASC analysis. (A) Gating strategy for skin T cells. (B) Shown are the representative dot plots and percentageS of CD4^+^ T_RM_ (CD4^+^CD69^+^CD103^+^) cells within CD4^+^ cells in psoriatic skin. (C) Shown are the representative dot plots and percentages of CD8^+^ T_RM_ (CD8^+^CD69^+^CD103^+^) cells within CD8^+^ cells. (D) Representating images of the immunofluorescent staining of CD8^+^CD103^+^ cells in psoriatic skin (red: CD103, green: CD8, 400×, Scale bar: 100 µm). Data are presented as mean ± SD (n = 5–8 mice/group, ^*^ *p* *< *0.05 and ^**^ *p* *< *0.01). One representative of three independent experiments is shown.

Given that high humidity promoted IMQ‐induced recurrence of psoriasis, we asked whether high humidity would affect the skin‐resident memory T cells. Skin was digested and cutaneous single cells were isolated and stained to quantify CD4^+^ T_RM_ (CD4^+^CD69^+^CD103^+^) and CD8^+^ T_RM_ (CD8^+^CD69^+^CD103^+^) cells. Figure 2A displayed the FACS gating strategies for skin T cells. As shown in Figure 2B,C, a substantial number of CD4^+^ and CD8^+^ T_RM_ cells resided in the skin of mice with IMQ‐induced psoriasis recurrence. High humidity significantly increased the percentage of CD8^+^ T_RM_ cells, with no effects on that of CD4^+^ T_RM_ cells. Furthermore, immunofluorescent staining of skin revealed that high humidity augmented the number of CD8^+^CD103^+^ T_RM_ cells, which were located predominantly in the epidermis (Figure 2D). These data suggest that high humidity aggravates psoriasis relapse by promoting CD8^+^ T_RM_ formation.

### 2.3 High‐Humidity Environment Upregulates the Expression of IL‐15Rα in Keratinocytes

**FIGURE 3:**
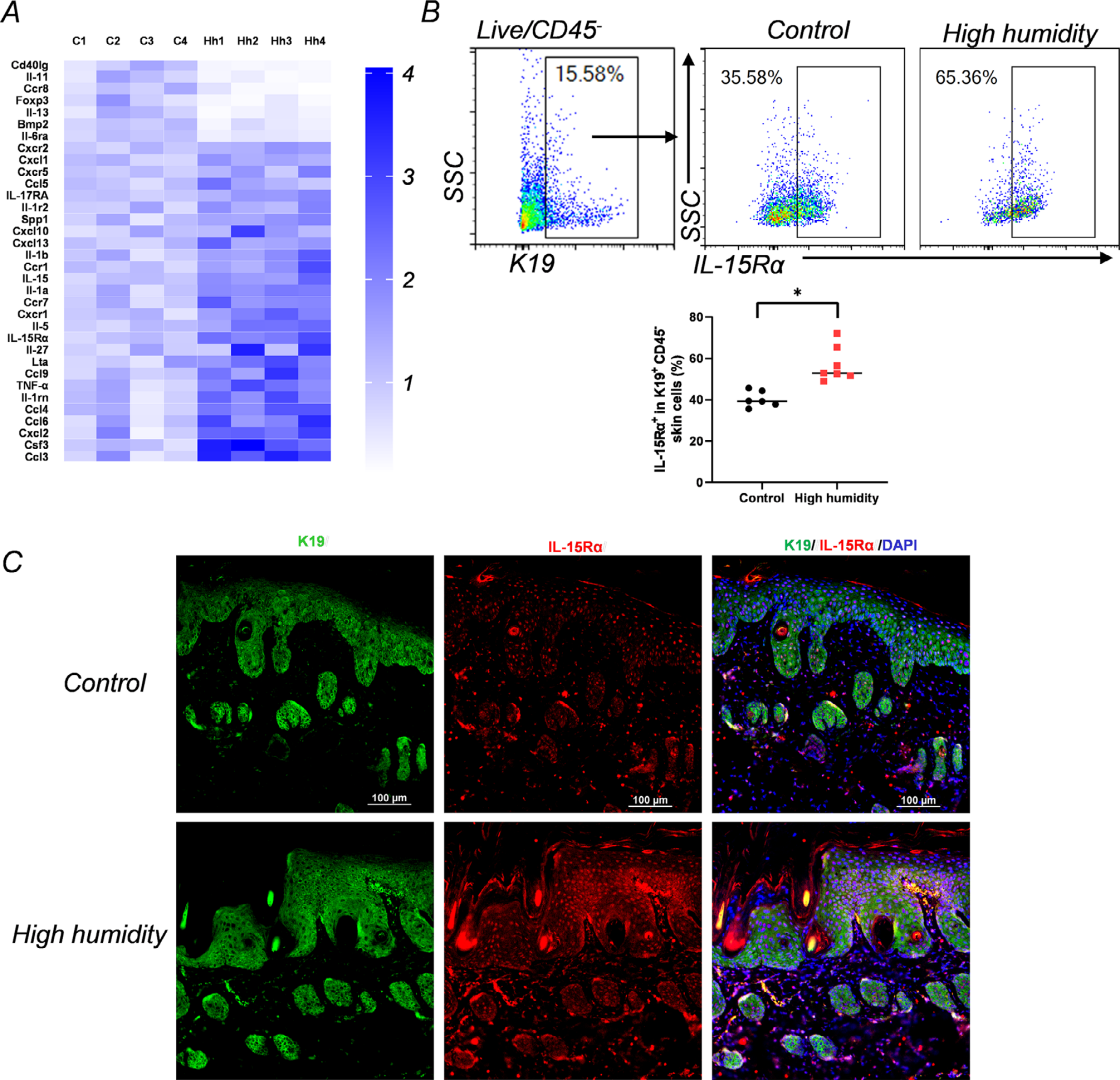
High humidity upregulates the expression of IL‐15Rα in keratinocytes. (A) A heatmap of PCR array analysis showing mRNA expression of inflammation‐related genes in the skin of mice with psoriasis relapse. (*n* = 4 mice/group). (B) The representative dot plots and percentages of IL‐15Rα^+^ cells within the keratinocyte population (CD45^−^K19^+^). (C) Immunofluorescence staining of K19^+^IL‐15Rα^+^ cells in the psoriatic skin (red: IL‐15Rα, green: k19, 200×,Scale bar: 50 µm). Data are presented as mean ± SD from two independent experiments (n = 6–7 mice/group, ^*^ *p* *< *0.05 and ^**^ *p* *< *0.01).

We first determined the cutaneous expressions of cytokines and chemokines using the inflammatory cytokine and receptor PCR arrays, with primers pre‐embedded in 96‐well plates. A total of 107 common genes were detected. As shown in Figure 3A, high humidity disrupted the balance of chemokines/chemokine receptors and cytokines/cytokine receptors by downregulating the expression of CD40Ig, IL‐11, CCR8, FoxP3, IL‐13 (Fold change ≥1.5, *p* < 0.05) and upregulating the expression of BMP2, IL‐6Ra, CXCR2, CXCL1, CXCR5, CCL5, IL‐17RA, IL‐1R2, SPP1, CXCL10, CXCL13, IL‐1b, CCR1, IL‐15, IL‐1a, CCR7, CXCR1, IL‐5, IL‐15Rα, IL‐27, Ltα, CCl9, TNF‐α, IL‐1R2, CCL4, CCL6, CXCL2, CSF3 and CCL3 (Fold change ≥1.5, *p* < 0.05). We were focused on IL‐15/IL‐15Rα since IL‐15 or IL‐15Rα is critically involved in the development and survival of tissue‐resident memory T cells [31]. FASC analysis further showed that high humidity significantly upregulated the IL‐15Rα expression in keratinocytes of mice with psoriasis relapse (Figure 3B). Based on immunofluorescent staining, we also found that IL‐15Rα was mainly expressed in K19^+^ keratinocytes, while high humidity exposure upregulated IL‐15Rα expression in keratinocytes compared to that in control mice (Figure 3C). Overall, our findings suggest that exacerbation of IMQ‐induced psoriasis relapse by high humidity may be connected to higher IL‐15Rα expression on keratinocytes.

### 2.4 An Increase in IL‐15Rα on Keratinocytes is Responsible for the Exacerbation of Psoriasis Relapse by High Humidity (Hh)

**FIGURE 4:**
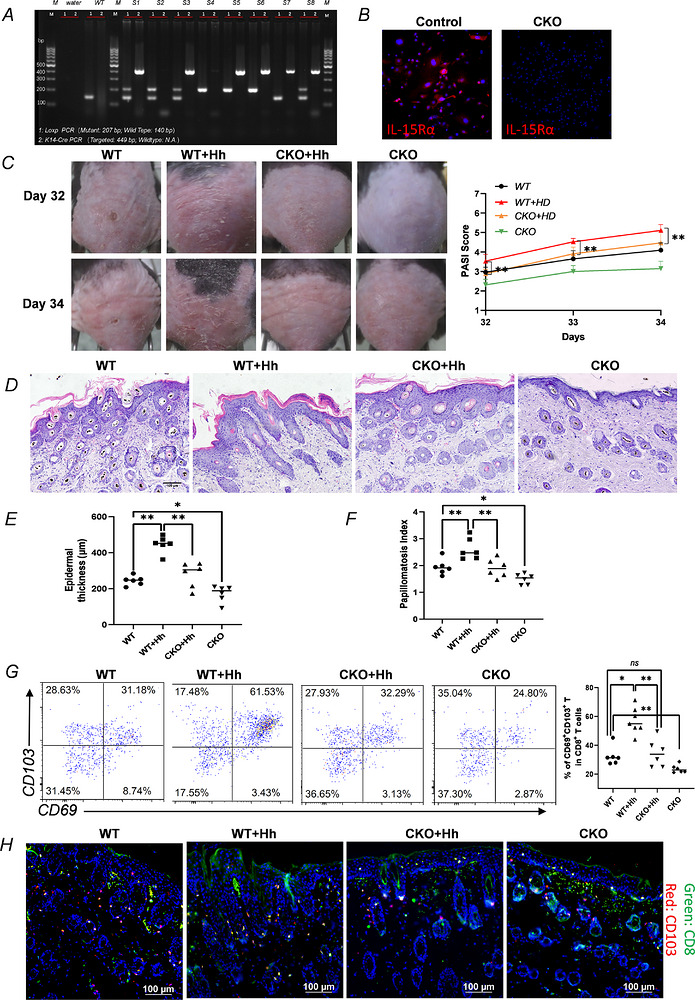
High humidity‐induced psoriasis relapse and CD8^+^ T_RM_ accumulation are dependent on IL‐15Rα expression in keratinocytes. (A) Agarose gel electrophoresis of PCR products from tail samples of keratinocyte‐specific IL‐15Rα knockout (CKO) mice (Loxp PCR‐Wild type: 140 bp, Mutant: 207 bp; K14‐Cre PCR‐ Targeted: 449 bp, Wild type: N.A.). (B) Primary keratinocytes from wild type mice (WT Control) or IL‐15Rα.Loxp/loxp‐K14‐Cre (CKO) mice were isolated and treated with the cytokine cocktail for 48 h. IL‐15Rα expression in CKO or WT control mice was detected by immunofluorescent staining. (C) WT or CKO mice kept under normal control or high humidity environment were challenged with IMQ to induce psoriasis relapse as indicated in Figure 1A. Shown are representative images and PASI scores of skin lesions. (D‐F) Representative images of H&E staining of psoriatic skin (200×, Scale bar: 100 µm), the epidermal thickness and papillomatosis index based on H&E staining. (G) Shown are the representative dot plots and percentages of CD8^+^ T_RM_ (CD8^+^CD69^+^CD103^+^) cells within CD8^+^ cells from psoriatic skin. (H) Representative images of immunofluorescent staining of CD8^+^CD103^+^ cells in the psoriatic skin (red: CD103, green: CD8, 400×, Scale bar: 100 µm). Data are presented as mean ± SD from two independent experiments (n = 6 mice/group).

IL‐15 is a critical cytokine for the formation and maintenance of CD8^+^ memory T cells. Its function requires binding to and signaling through its specific receptor IL‐15Rα, while IL‐15Rα on macrophages in turn binds IL‐15 and presents it to T cells. Here, we asked whether IL‐15Rα on keratinocytes contributed to the psoriasis relapse aggravated by high humidity. We first generated keratinocyte‐specific IL‐15Rα conditional knockout (CKO) mice by cross‐breeding of IL‐15Rα.Loxp/Loxp and K14‐Cre mice (Figure 4A). Primary keratinocytes from CKO mice activated in vitro were proved to lack IL‐15Rα (Figure 4B). Keratinocyte‐specific IL‐15Rα CKO mice or WT controls were challenged with IMQ to induce psoriasis relapse as indicated in Figure 1A. As shown in Figure 4C, keratinocyte‐specific IL‐15Rα deficiency significantly ameliorated the psoriatic skin lesions compared to WT mice. Moreover, lack of IL‐15Rα in keratinocytes attenuated high humidity (Hh)‐induced psoriatic skin lesions compared to control groups with Hh. H&E staining also confirmed that IL‐15Rα‐deficiency in keratinocytes lessened epidermal hyperplasia and hyperkeratosis compared to WT group, while high humidity failed to aggravate epidermal hyperplasia and hyperkeratosis in keratinocyte‐specific IL‐15Rα CKO mice (Figure 4D–F). To further determine whether upregulation of skin T_RM_ cells by high humidity was dependent on IL‐15Rα in keratinocytes, we compared the skin CD8^+^ T_RM_ population in IL‐15Rα CKO and WT mice with(out) high humidity by FASC analysis and immunofluorescent staining. As shown in Figure 4G,H, keratinocyte‐specific IL‐15Rα‐knockout significantly reduced the percentage of CD8^+^CD69^+^CD103^+^ cells in the psoriatic skin compare to WT mice. Moreover, high humidity failed to upregulate CD8^+^CD69^+^CD103^+^ population in keratinocyte‐specific IL‐15Rα CKO mice compared to WT mice kept in high humidity. Importantly, blocking IL‐15/IL‐15Rα interaction with soluble sIL‐15Rα or neutralizing anti‐IL‐15 Ab largely reversed the skin lesions and pathology induced by high humidity in mice with psoriasis relapse (Figure S1A–E) and reduced skin CD8^+^ T_RM_ cells as well (Figure S1F). These findings suggest that IL‐15Rα on keratinocytes contributes, at least in part, to high humidity‐induced psoriasis relapse and generation of skin CD8^+^ T_RM_ cells.

### 2.5 IL‐15Rα‐Expressing Keratinocytes Promote CD8^+^ T_RM_ Generation In Vitro

**FIGURE 5:**
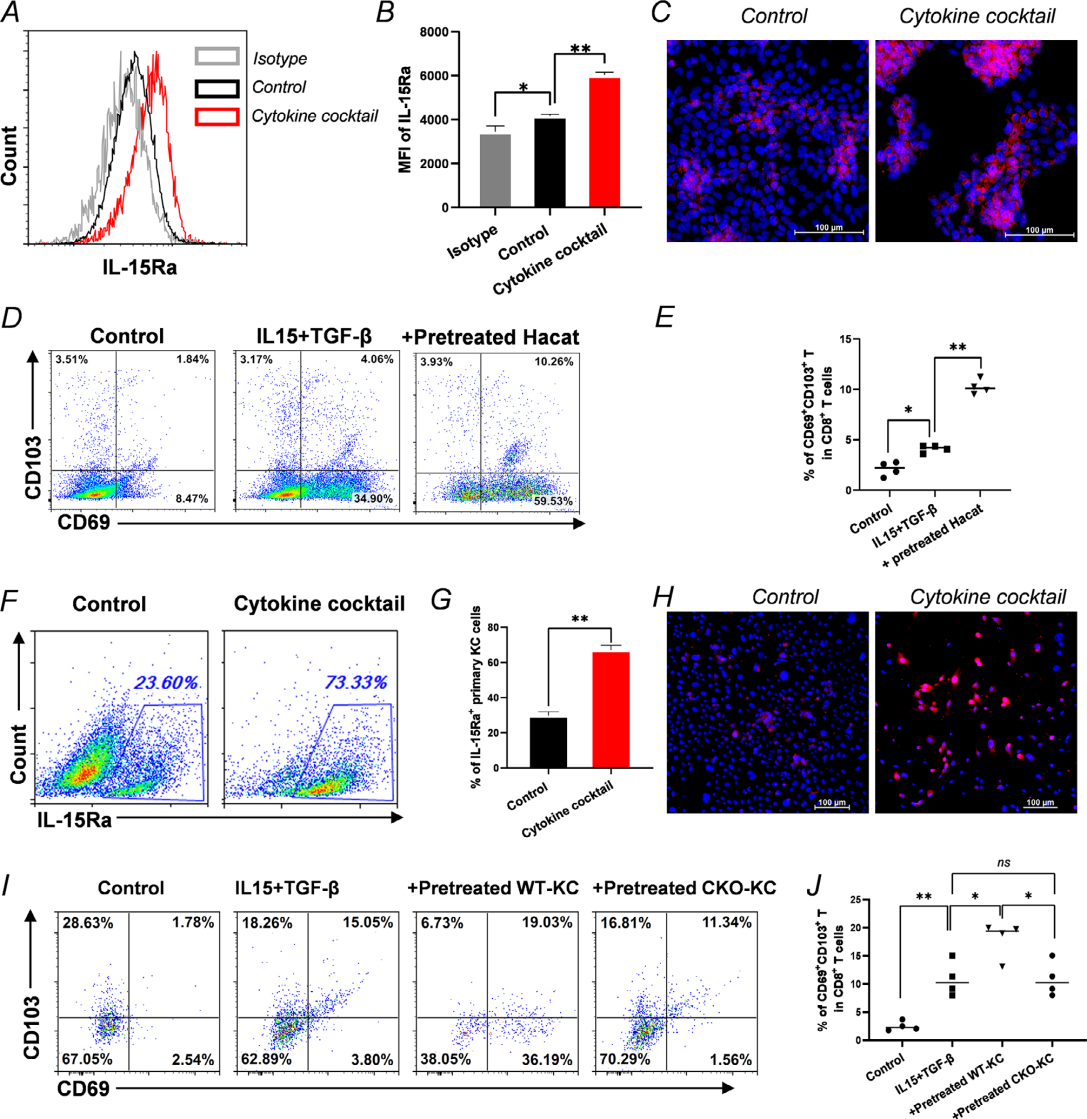
Keratinocytes expressing IL‐15Rα promote T_RM_ generation in vitro. (A–C) Human Hacat cells were first treated with cytokine cocktail (IL‐17A 20 ng/mL, TNF‐α 20 ng/mL, IL‐22 10 ng/mL, and IFN‐γ 20 ng/mL) for 48 h. IL‐15Rα expression by Hacat cells was analyzed via FASC, as represented by mean fluorescence intensity (MFI) and immunofluorescence staining. (D, E) Human PBMCs were cultured with IL‐2 and IL‐15 for three days, followed by medium renewal with addition of TGF‐β1 for additional three days. In coculture group, Hacat cells pretreated with cytokine cocktail were added to PBMC culture. The frequency of CD8^+^CD69^+^CD103^+^ T cells was determined by FACS. (F) Primary murine keratinocytes were cultured for differentiation for 4 days. The differentiated cells were then treated with the cytokine cocktail for 48 h, and IL‐15Rα expression in keratinocytes was analyzed by FACS. (G) The frequency of IL‐15Rα^+^ cells in keratinocytes. (H) Immunofluorescent staining of IL‐15Rα in keratinocytes. (I, J) Splenocytes from C57BL/6 mice were treated as described in D to induce T_RM_ cells. Primary murine keratinocytes from WT or CKO mice were pretreated with cytokine cocktail and then cocultured with the splenocytes. CD8^+^CD69^+^CD103^+^ T cell frequency was determined by FACS on day 6. Data are presented as mean ±SD (n = 4/group, ^*^p < 0.05 and ^**^ *p < *0.01). One representative of three independent experiments is shown.

We then validated these effects of IL‐15Rα on CD8^+^ T_RM_ generation using an in vitro coculture model. First, we determined the expression of IL‐15Rα in both Hacat cells (a human cell line of keratinocytes) and primary mouse keratinocytes. As shown in Figure 5A–C, cytokine cocktail (TNF‐α, IL‐17A, IL‐22, and IFN‐γ) treatment significantly upregulated IL‐15Rα expression on Hacat cells. As expected, IL‐15+TGFβ also induced CD8^+^CD69^+^CD103^+^ T_RM_ cells from human PBMCs. Importantly, addition of the cocktail pretreated Hacat cells to the PBMC culture further promoted the differentiation of CD8^+^CD69^+^CD103^+^ T_RM_ (Figure 5D,E). Similarly, the cytokine cocktail increased IL‐15Rα expression on primary mouse keratinocytes (Figure 5F–H). However, the cocktail pretreated keratinocytes from IL‐15Rα CKO mice (Pretreated CKO‐KC) failed to augment the percentage of CD8^+^CD69^+^CD103^+^ T_RM_ cells (Figure 5I,J) compared to those from WT mice (Pretreated WT‐KC). In summary, these data suggest that activated keratinocytes expressing IL‐15Rα promote in vitro generation of CD8^+^ T_RM_ cells in a IL‐15Rα‐dependent manner.

### 2.6 High‐Humidity Environment Alters the Skin Microbiome Diversity in IMQ‐Induced Murine Models of Psoriasis Relapse

**FIGURE 6:**
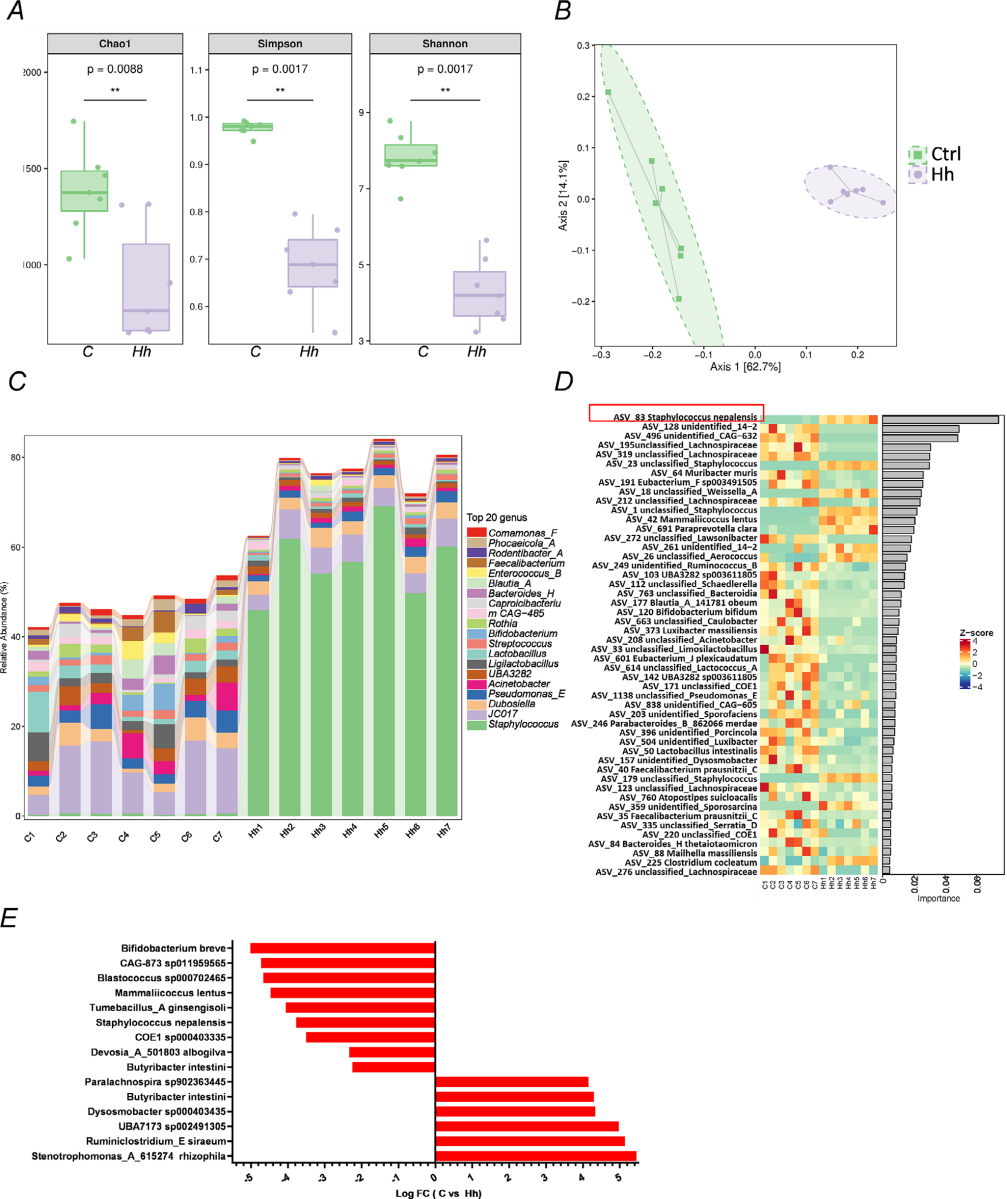
High humidity environment alters the skin microbiome diversity in IMQ‐induced mouse models of psoriasis relapse. Skin swab samples from the back of the psoriatic mice were collected and the microbial community characteristics were detected by 16S rDNA sequencing. (A) Boxplots of alpha‐diversity indices for the high humidity and normal control groups, showing (from left to right) Chao1, Simpson and Shannon indices. (B) Boxplots of β‐diversity indices for the high humidity and normal control groups, as determined by PCoA analysis. (C) Analysis of differential species at a genus level. (D) Analysis of biomarker species using random forest analysis. (E) Different species identified and annotated at the species levels.

Alteration of the skin microbiota influences the onset and progression of psoriasis. Although *Staphylococcus aureus* was positively associated with psoriatic skin inflammation, the abundance of *Staphylococcus epidermidis* or *propionibacterium* was much lower in psoriatic skin lesions than in healthy skin [32]. We then determined the effects of a high‐humidity environment on the composition of the skin microbiome in mice with psoriasis relapse. As shown in Figure 6A, high humidity significantly reduced the diversity of the skin microbial community, as evidenced by decreased alpha diversity indices, including community richness (Chao1), evenness (Simpson diversity index), and overall diversity (Shannon index). Moreover, 16S rDNA sequencing analysis revealed significant differences in the beta diversity of the skin microbiome between the high‐humidity and control group of mice with psoriasis recurrence. The principal coordinates analysis (PcoA) showed distinct clustering patterns between the two groups on the 2D scatter plots, indicating that a high‐humidity environment had a notable impact on the skin microbiome composition (Figure 6B). In addition, species composition analysis also revealed significant differences in the microbial composition between the two groups, with high humidity group exhibiting increased abundance of *Staphylococcus* genus (Figure 6C). Further, random forest analysis identified key differential species between the two groups. Figure 6D showed the top 100 ASVs ranked by the importance according to the random forest analysis. Some, but not all, ASVs were annotated to the levels of specific species. The highest scoring of ASV was ASV83, which was annotated as *Staphylococcus nepalensis* based on the database of Greengenes. Moreover, Metagenome Seq analysis identified specific microbial genes that were distinct in abundance, with some genes being also annotated to specific species (Figure 6E).

### 2.7 *Staphylococcus nepalensis (S. nepalensis)* Colonization Exacerbates the Recurrence of IMQ‐Induced Murine Psoriasis

**FIGURE 7:**
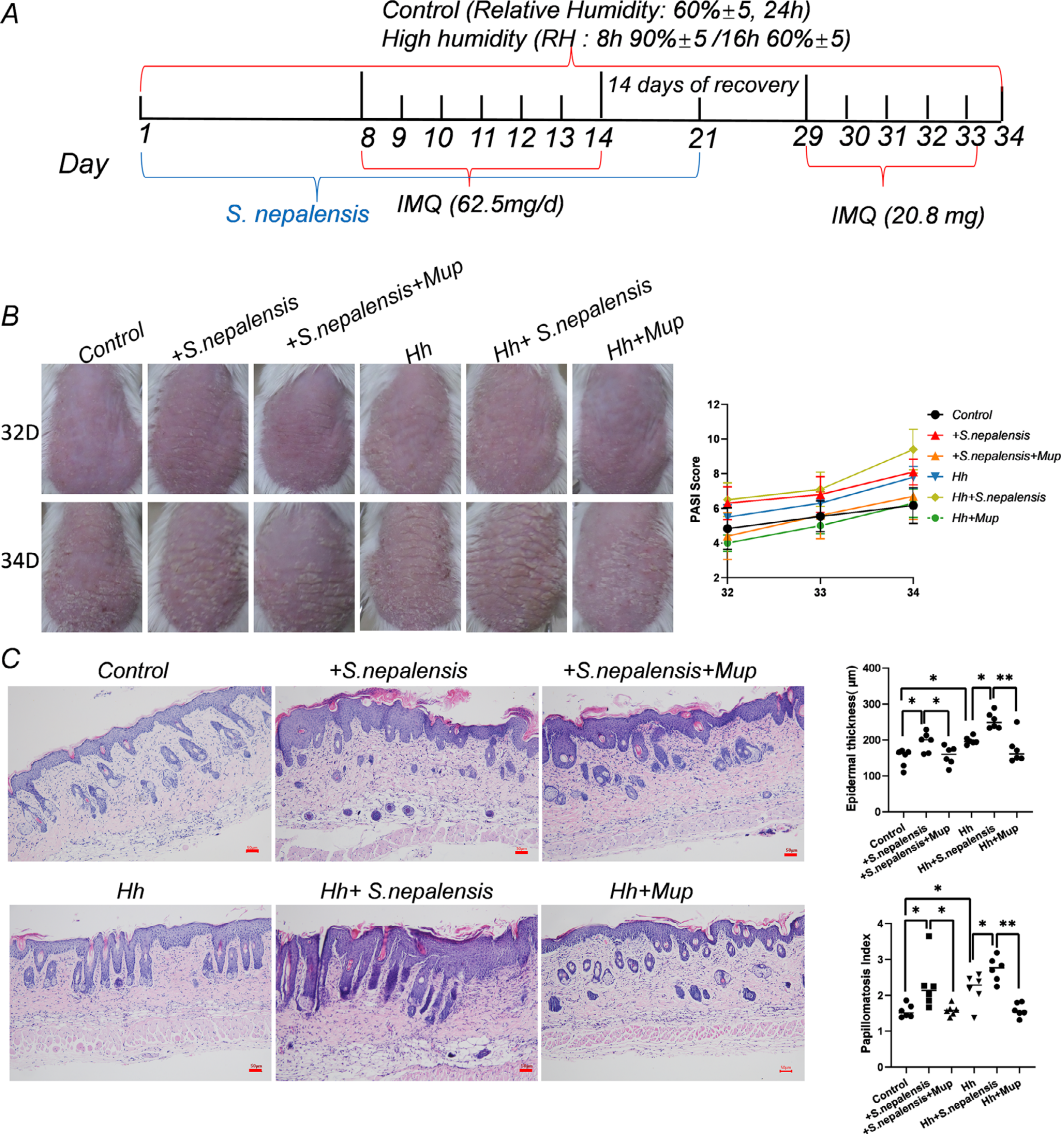
*S. nepalensis* colonization exacerbates the recurrence of IMQ‐induced psoriasis. (A) Experimental scheme of *S. nepalensis* and/or *Mupirocin* treatment as a potentially positive or negative intervention for the psoriasis relapse. (B) Representative images and PASI scores of skin lesions during relapse phase (200×, Scale bar: 100 µm). (C) Images of H&E staining of the psoriatic skin, the epidermal thickness and papillomatosis indices based on H&E staining. (n = 6, ^**^ *p* *< *0.01). One representative of three independent experiments is shown.

Since the highest scoring of ASV under high‐humidity condition was ASV83 that was annotated as *S. nepalensis*, we then assessed the effects of skin *S. nepalensis* colonization on the relapse of psoriasis (Figure 7A). We found severer skin lesions and higher PASI scores in *S. nepalensis‐*colonized mice than in control mice (Figure 7B). As expected, high humidity (Hh) worsened the skin lesions compared to control mice, while mupirocin (Mup), a sensitive antibiotic, reversed the skin lesions induced by high humidity (Figure 7B). Interestingly, Hh plus *S. nepalensis* further increased skin lesions and PASI scores on day 34 compared to Hh or *S. nepalensis* alone, whereas treatment with both Mup and *S. nepalensis* significantly reduced the lesions and scores compared to *S. nepalensis* alone. Inhibition tests in vitro showed strong inhibitory effects of mupirocin on the growth of *S. nepalensis* (Figure S2). Moreover, H&E staining also confirmed that *S. nepalensis‐*colonized mice exhibited higher epidermal thickness and papillomatosis indexes compared with control mice, while mupirocin treatment alleviated the epidermal hyperplasia and hyperkeratosis compared to high‐humidity group without mupirocin (Figure 7C). Similarly, Hh plus *S. nepalensis* further increased epidermal thickness and papillomatosis indexes compared to either Hh or *S. nepalensis* alone.

To determine whether the observed effects are due to bacterial infection‐induced non‐specific inflammation, we inoculated mice in the IMQ‐induced psoriasis model with *S. nepalensis* or *S. epidermidis—*a common commensal skin bacterium serving as a control. As shown in Figure S3, compared to mice colonized with S. epidermidis, S. nepalensis‐colonized mice showed much more severe skin lesions, as evidenced by higher PASI scores, increased epidermal thickening, and enhanced hyperkeratosis. Flow cytometric analysis further demonstrated that S. nepalensis colonization specifically promoted the accumulation of CD103^+^CD69^+^ T_RM_ cells and the expression of IL‐15Rα on keratinocytes, whereas S. epidermidis had no effects, confirming the strain‐specific proinflammatory role of S. nepalensis in the pathogenesis of psoriasis.

### 2.8 *S. nepalensis* Colonization Upregulates the IL‐15Rα Expression on Keratinocytes and Increases CD8^+^ T_RM_ Accumulation in the Skin of Mice With Psoriasis Relapse

**FIGURE 8:**
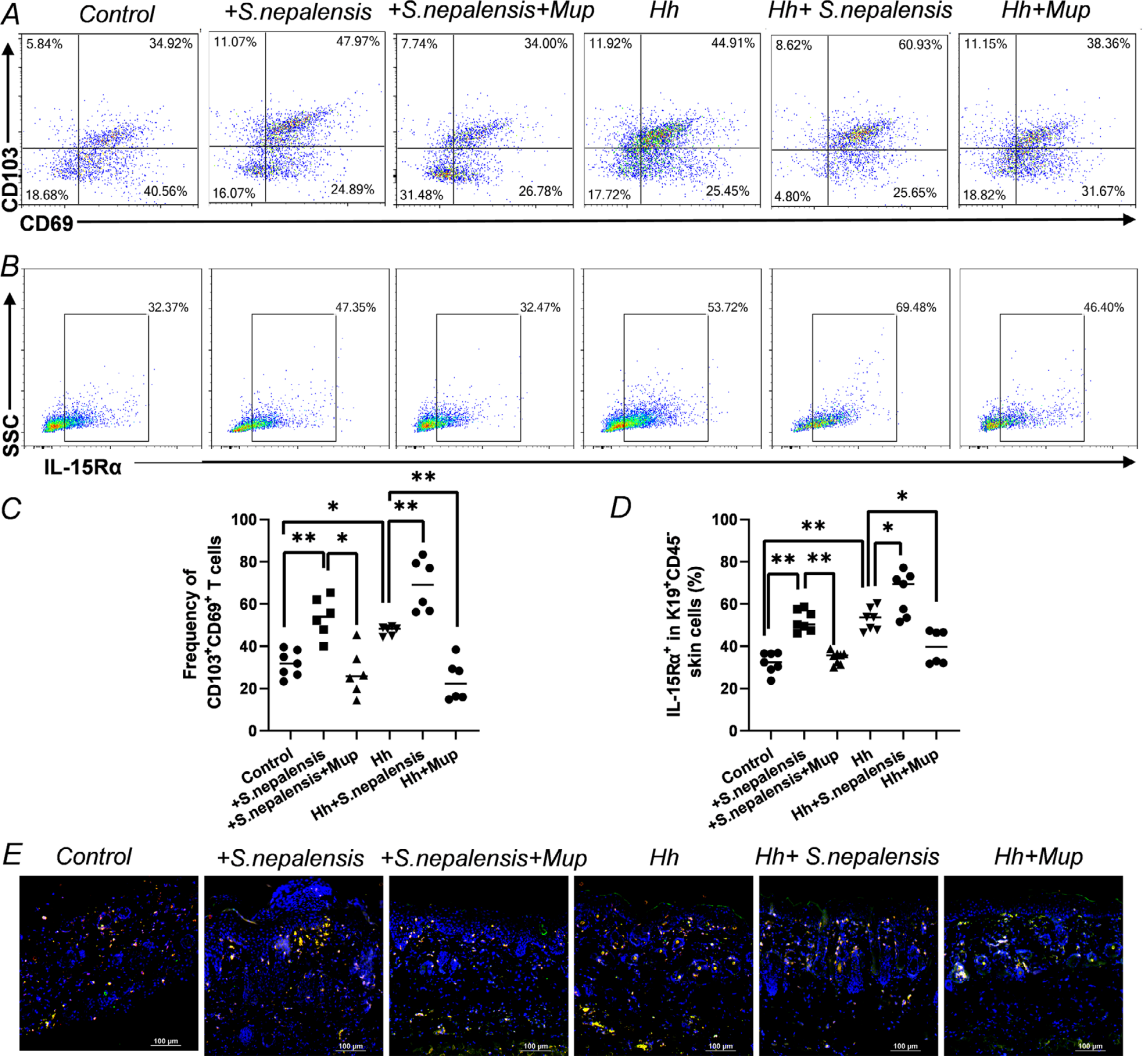
*S. nepalensis* colonization upregulates CD8^+^ T_RM_ accumulation and IL‐15Rα expression in the skin. (A) Representative dot plots and percentages of CD8^+^ T_RM_ (CD8^+^CD69^+^CD103^+^) cells within CD8^+^ T cells in psoriatic skin, as analyzed by FACS. (B) The representative dot plots and percentages of IL‐15Rα^+^ cells within the keratinocyte population (CD45^−^K19^+^) in the skin. (C) The scatter plots showing the average percentages of CD8^+^ T_RM_ cells and statistical significance. (D) The scatter plots showing the average percentages of IL‐15Rα^+^ cells within the keratinocyte population and statistical significance. (E) Representative images of the immunofluorescent staining of CD8^+^CD103^+^ cells in the skin sections (red: CD103, green: CD8, 400×, Scale bar: 100 µm). Data are presented as mean ± SD from two independent experiments (n = 6 mice/group).

To further understand mechanisms, we evaluated the skin T_RM_ accumulation and IL‐15Rα expression on keratinocytes by FACS analysis after the colonization of *S. nepalensis* or mupirocin treatment. The results showed that *S. nepalensis*‐colonized mice had more CD8^+^ T_RM_ cells than control mice (Figure 8A and C) and that *S. nepalensis* colonization increased IL‐15Rα expression on keratinocytes (Figure 8B and D). However, compared with high‐humidity alone group, addition of mupirocin (Mup) significantly reduced IL‐15Rα expression on keratinocytes (Figure 8B) and decreased the frequency of skin CD8^+^ T_RM_ cells (Figure 8A). Moreover, immunofluorescence experiments confirmed that more CD8^+^CD103^+^ T_RM_ cells were observed in the psoriatic skin of *S. nepalensis*‐colonized mice or mice with Hh than that of control mice (Figure 8E). However, S. nepalensis‐colonization plus Mup largely abolished the effects of the colonization alone. Taken together, these findings suggest that *S. nepalensis* colonization in skin increases IL‐15Rα expression on keratinocytes and skin CD8^+^ T_RM_ cells, whereas suppression of *S. nepalensis* by an antibiotic reverses these processes induced by high humidity.

### 2.9 The Supernatant of *S. nepalensis* Culture Upregulates the Expression of IL‐15Rα on Keratinocytes In Vitro by Activating Their NF‐κB Signaling Pathway

Given that the abundance of *Staphylococcus* genus significantly changed under high‐humidity environment, and that *S. nepalensis* had the highest disease contribution in random forest analysis, we then examined the direct effects of *S. nepalensis* on IL‐15Rα expression on keratinocytes in vitro. As shown in Figure S4, 5% *S. nepalensis* supernatant significantly increased IL‐15Rα expression on normal Hacat cells, while both 2.5% and 5% *S. nepalensis* supernatant did so on Hacat cells pre‐treated with cytokine cocktail (Figure S4). These findings indicate that high humidity may influence the pathological progression of psoriasis by altering the skin microbiome composition, which in turn promotes IL‐15Rα expression on keratinocytes. For mechanistic studies, we further examined NFκB signaling in keratinocytes in vitro, since it is critical for the overall gene expression of IL‐15Rα [33] and particularly IL‐15Rα expression on keratinocytes [34]. Based on Western blotting analysis, we found that 5% S. nepalensis enhanced phosphorated‐IKBα (p‐IKBα) and p‐P65 protein expression in Hacat cells (Figure S5A,B). On the other hand, the nepalensis‐mediated increase in IL‐15Rα expression on keratinocytes was largely abolished by blocking their NFκB signaling with BAY11‐7082 (Figure S5C), indicating that *S. nepalensis* upregulates IL‐15Rα expression on keratinocytes by activating their NFκB signaling.

### 2.10 *S. nepalensis*‐Specific Metabolite, ADMA, Activates NF‐κB Signaling and Exacerbates Psoriasis‐Like Skin Inflammation

**FIGURE 9:**
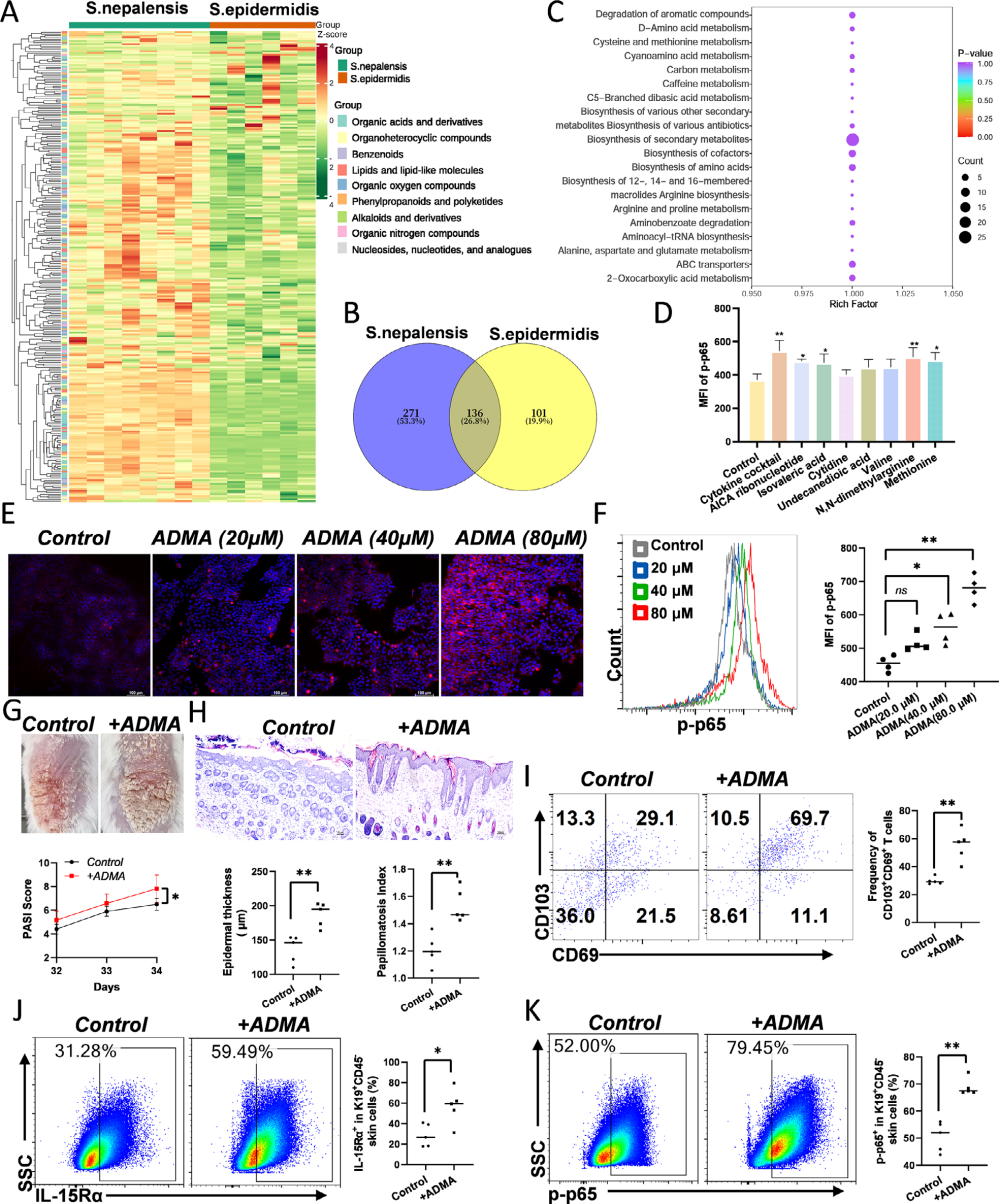
*S*. nepalensis‐specific metabolite ADMA activates NF‐κB signaling and exacerbates psoriasis‐like inflammation. (A) A heatmap of differential metabolites in *S*. nepalensis and S. epidermidis cultures (n = 6–8). (B) Venn diagram showing unique (271) and shared (136) metabolites between S. nepalensis and S. epidermidis. (C) Enrichment analysis of metabolic pathways associated with S. nepalensis‐specific metabolites. (D) Quantification of p‐p65 based on mean fluorescence intensity (MFI) in HaCaT cells treated with candidate metabolites at 10 µm (n = 4). (E) Immunofluorescence staining of p‐p65 in HaCaT cells treated with 20, 40, or 80 µm ADMA (200×, scale bar:100 µm). (F) Flow cytometric histograms and MFI quantification of p‐p65 in HaCaT cells treated with ADMA (n = 4). (G) Representative photos of skin lesions and PASI scores of IMQ‐induced psoriatic mice treated with ADMA (n = 5). (H) H&E staining (200×, scale bar: 100 µm) and quantification of epidermal thickness and papillomatosis indexes in ADMA‐treated mice. (I) Flow cytometric analysis of CD103^+^CD69^+^ T_RM_ cells (gated on CD8^+^ T cells) in lesional skin (n = 5). (J) Flow cytometric analysis of IL‐15Rα^+^ cells within keratinocyte population (CD45^−^K19^+^) (n = 5). (K) Flow cytometric analysis of p‐p65^+^ cells in keratinocytes (n = 5). Data are presented as mean ± SD. ^*^ *p* < 0.05, ^**^ *p* < 0.01 vs. Control based on one‐way ANOVA or Student's *t*‐test.

Untargeted metabolomic analysis of bacterial supernatants identified 271 metabolites uniquely present in S. nepalensis but absent in S. epidermidis (Figure 9A,B). Functional enrichment analysis revealed that these S. nepalensis‐specific metabolites were significantly associated with molecular pathways linked to NF‐κB signaling, including arginine and proline metabolism (Figure 9C). To identify the functional metabolites, we screened the candidate metabolites from these enriched pathways by treating HaCaT cells and assessing p‐p65 expression via flow cytometry. From this screening, asymmetric dimethylarginine (ADMA) was selected as a top candidate for its potential ability to induce p‐p65 expression (Figure 9D). ADMA treatment (20–80 µm) in vitro dose‐dependently increased p‐p65 levels and promoted its nuclear translocation in HaCaT keratinocytes, as also confirmed by flow cytometry and immunofluorescence (Figure 9E,F). Furthermore, intradermal injection of ADMA significantly exacerbated the disease severity in an IMQ‐induced psoriasis relapse model, as evidenced by elevated PASI scores (Figure 9G), increased epidermal thickness, and papillomatosis index (Figure 9H). Flow cytometry demonstrated that ADMA treatment markedly increased the frequency of skin CD103^+^CD69^+^ tissue‐resident memory CD8^+^ T (T_RM_) cells (Figure 9I) and IL‐15Rα^+^ keratinocytes (Figure 9J), while elevating p‐p65 expression (Figure 9K) in keratinocytes. Collectively, these results demonstrated that ADMA, a metabolite uniquely enriched in culture supernatant of S. nepalensis, directly activates the NF‐κB pathway in keratinocytes and promotes T_RM_ cell accumulation, thereby worsening psoriasiform inflammation.

### 2.11 High Humidity Aggravates Human Skin Lesions in Humanized Mice Transplanted With the Lesional Skin From Psoriasis Patients

**FIGURE 10:**
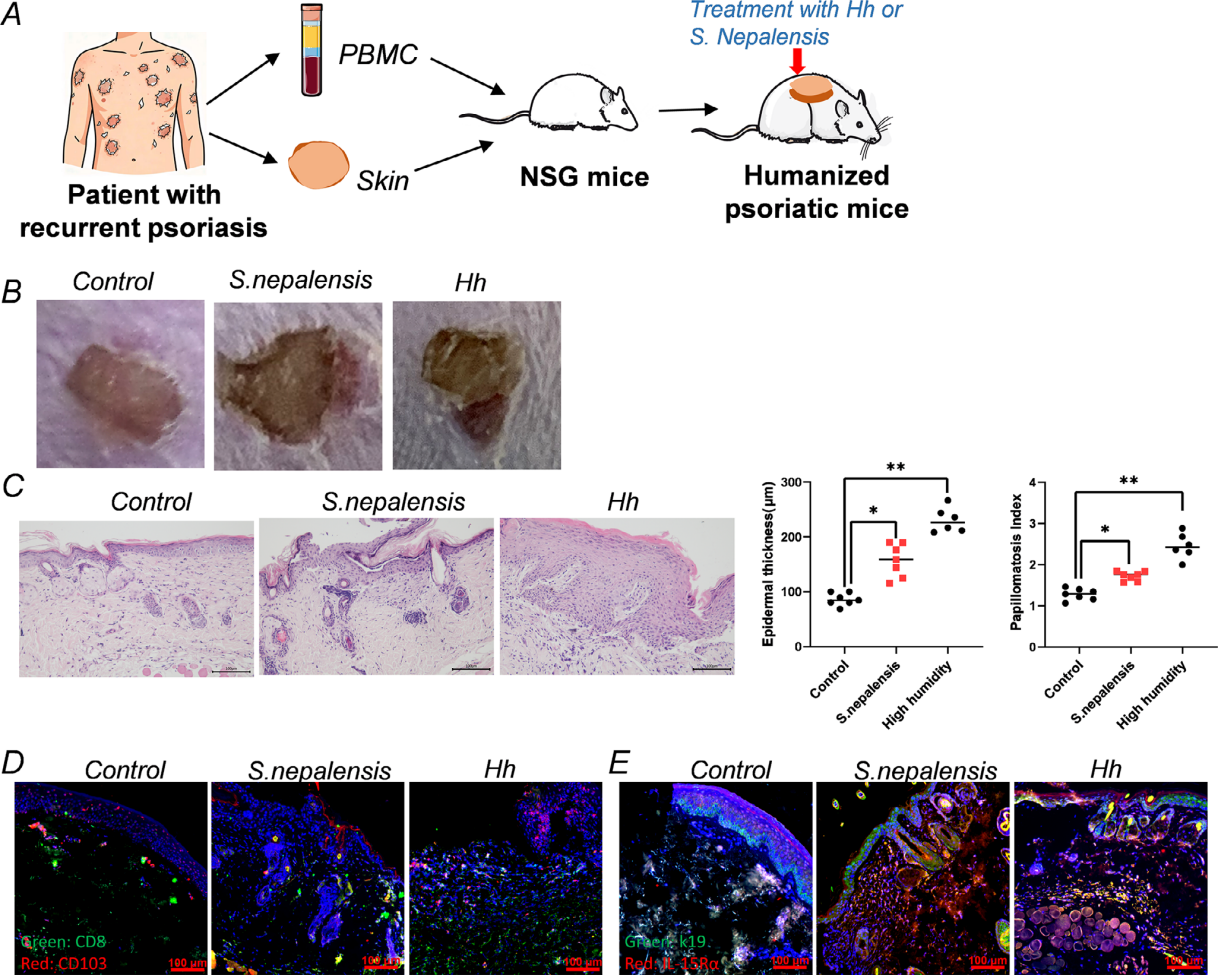
High humidity aggravates psoriatic human skin lesions in humanized mice. The moderately lesional skin from psoriatic patients was transplanted to NSG mice that received PBMCs from the same patients. The recipient mice were either subjected to high humidity (**Hh**) environment or treated topically with S. *nepalensis* for ten days. (A) The procedure of humanized mouse models with skin grafts derived from psoriatic patients. (B) The representing images of transplanted human skin in recipient mice were shown. (C) H&E staining of human skin grafted in NSG mice (200×, Scale bar:100 µm) with epidermal thickness and papillomatosis index. (D) Immunofluorescent staining of grafted human skin for CD8 (green) and CD103 (red) (200×, Scale bar:100 µm). (E) Immunofluorescent staining of grafted human skin for K19 (green) and IL‐15Rα (red) (200×, Scale bar:100 µm). Data are presented as mean ±SD, n = 6–7 human skin samples in mice per group, ^*^ *p < *0.05 and ^**^ *p* *< *0.01.

To confirm the effects of high humidity on human psoriasis, moderately lesional skin from psoriatic patients was transplanted to NSG mice that received PBMCs from the same patients (Figure 10A). The recipient mice were either subjected to a high‐humidity (Hh) environment or treated topically with S. *nepalensis* for ten days. The representing images of transplanted human skin in recipient mice are shown in Figure 10B. The lesions of skin grafts in recipient mice looked much worse in mice subjected to high humidity or applied with S. *nepalensis* than in control mice ten days post‐transplantation. H&E staining demonstrated that the epidermal thickness or papillomatosis index of skin grafts in recipient mice subjected to Hh or S. *nepalensis* was much greater than that of control mice (Figure 10C). Furthermore, to determine if Hh also affects memory CD8^+^ T_RM_ accumulation in the human skin derived from psoriasis patients, we examined CD8^+^ T_RM_ cells via immunofluorescent staining of the human skin graft. We found that treatment with Hh or S. *nepalensis* increased CD8^+^CD103^+^ T_RM_ cells in grafted human skin, while these memory cells were barely detectable in control mice (Figure 10D). In addition, immunofluorescent staining of psoriatic human skin grafts confirmed that either *S. nepalensis* colonization or exposure to high humidity (Hh) significantly upregulated the expression of IL‐15Rα on epidermal keratinocytes (K19^+^ cells) compared to the control group (Figure 10E). This finding mirrors our observation in the wild‐type mouse model and suggests that *S. nepalensis* specifically drives the expansion of IL‐15Rα^+^ keratinocytes in human skin. Together, these data demonstrate that the expansion of IL‐15Rα^+^ keratinocytes and induction CD8^+^ T_RM_ are a conserved human‐relevant mechanism underlying *S. nepalensis*‐mediated exacerbation of psoriasis.

## 3 Discussion

In this study, we evaluated the impacts of high humidity on the psoriatic skin inflammation and relapse of psoriasis using IMQ‐induced psoriatic mouse models. Our study yielded the following findings: (1) psoriatic mice housed in a high‐humidity environment were more prone to relapse, and the skin lesions were more severe upon recurrence; (2) high‐humidity exposure induced an elevated level of skin resident memory CD8^+^ T cells; (3) Keratinocyte‐specific IL‐15Rα upregulated by high humidity drove the accumulation of skin CD8^+^ T_RM_; (4) abnormality in skin microbiota induced by high humidity environment resulted in an increase of IL‐15Rα expression in keratinocytes and promoted the CD8^+^ T_RM_ formation. Moreover, high humidity also aggravated human skin lesions in humanized mice that received skin grafts from psoriatic patients. Collectively, our data demonstrated that a high‐humidity environment exacerbated the relapse of psoriasis by altering the skin microbiota and increasing the expression of IL‐15Rα on keratinocytes, which in turn promoted cutaneous CD8^+^ T_RM_ cell formation.

It has been reported that a high‐humidity environment exacerbates some allergic and autoimmune diseases, including arthritis [35], lupus nephritis [36], and asthma [37, 38]. However, a dry environment may also influence the skin barrier by rendering skin more sensitive and prone to inflammation [39]. The prevalence of atopic dermatitis has also been inversely related to environmental humidity [40]. On the other hand, low humidity appears to be associated with a high morbidity rate of psoriasis [10], while high humid condition may contribute to its low morbidity [11]. However, it remains unknown if humidity affects psoriasis relapse and whether its recurrence in turn impacts the overall morbidity rate. In the present study, we found that high humidity exacerbated the recurrence of psoriasis in IMQ‐induced psoriatic mice. Thus, our findings did not necessarily challenge the traditional dogma, but instead provided novel insights into how humidity impacts psoriasis relapse.

Although the exact mechanisms underlying psoriasis recurrence are not fully understood, it is believed that memory T cells are important for its relapse. Indeed, we previously demonstrated that skin CD8^+^ T_RM_ cells contributed to the relapse of IMQ‐induced psoriasis in wild‐type mice and in a humanized psoriatic mouse model as well, and that adoptive transfer of CD8^+^ T_CM_ cells promoted the psoriasis relapse [14, 41]. Here, we found that high‐humidity environment accelerated the recurrence of psoriasis in mice, accompanied by an increase in skin‐resident memory CD8^+^ T cells (T_RM_), indicating that high‐humidity environment may aggravate the recurrence of psoriasis by inducing CD8^+^ T_RM_ cells.

In the present study, high humidity significantly increased IL‐15Rα expression on keratinocytes, which was required for CD8^+^ T_RM_ cell formation in the psoriatic skin. Mackay, L., et al. found that IL‐15 deficiency reduced T_RM_ cells in skin and spleen. In addition, they found that IL‐15 production was restricted to radio‐resistant cells, such as skin keratinocytes and Langerhans cells, which are sufficient to support skin T_RM_ development and survival [17]. However, IL‐15 is barely secreted in the soluble form but bound to its specific receptor IL‐15Rα, especially under inflammatory conditions, meaning that IL‐15Rα can also function by presenting IL‐15 to T cells. Thus, both IL‐15 and IL‐15Rα knock‐out mice exhibited a reduction in memory CD8^+^ T cell and NK cells [42]. Furthermore, IL‐15 and IL‐15R IL‐15Rα needed to be co‐expressed by the same cells to trans‐present IL‐15 [43]. Mortier, E. et al. found that IL‐15Rα on macrophages supported the early conversion of antigen‐specific effector CD8^+^ T cells to memory T cells by cross‐presenting IL‐15 [18]. In addition, it was reported that keratinocytes from psoriatic mice also expressed surface IL‐15Rα [19]. All these findings implies that IL‐15Rα on keratinocytes or macrophages may support memory T cell formation by trans‐presenting IL‐15 in the context of psoriasis. However, sIL‐15Rα released by keratinocytes or even macrophages likely inhibited memory T cell development and psoriasiform skin inflammation [19]. We found that an inflammatory cytokine‐cocktail significantly upregulated IL‐15Rα expression on both primary mouse keratinocytes and Hacat cells (a human cell line), and that high humidity exposure aggravated the skin inflammation, accompanied by an increase in IL‐15Rα expression on keratinocytes. More importantly, either administration of IL‐15‐neutralizing Ab and soluble sIL‐15Rα, which neutralizes IL‐15Rα, or keratinocyte‐specific IL‐15Rα knock‐out alleviated psoriasis relapse and reduced the skin CD8^+^ T_RM_ accumulation. Keratinocytes promoted CD8^+^ T_RM_ development in vitro, which was dependent on IL‐15Rα on keratinocytes. Thus, high humidity exacerbated psoriasis recurrence by augmenting skin CD8^+^ T_RM_ through enhancing IL‐15Rα expression on keratinocytes.

High‐humidity environment has a great impact on the microbial ecology of the body [44, 45, 46, 47]. Guo, Y. et al. reported that high‐humidity environment significantly altered the colonic microbiota and fecal metabolome in mice and activated IL17/IL‐17R signaling in the colon [48]. In murine flu animal models, high‐humidity/temperature environment aggravated the intestinal inflammation by increasing Th17 cells while inhibiting Treg maturation [49]. Thus, environmental humidity may regulate the immune balance by altering the microbiota. However, there is little study concerning the effects of high humidity on skin microbiota. Skin microbiome profiling revealed significant differences between the psoriasis‐associated and healthy skin microbiota. Previous study showed that exacerbation of psoriasis was interconnected with epidermal colonization with *Streptococci, Malassezia or Staphylococcus* aureus [50]. Stimulation by Staphylococcus aureus or *Streptococcus* pyogenes enhanced IL‐17‐ and TNF‐α‐induced inflammatory responses in keratinocytes [51]. *Staphylococcus* aureus was positively associated with psoriatic skin lesions, while mice re‐colonized with *Staphylococcus* aureus demonstrated strong Th17 polarization [32]. Stimulation with heat‐killed *Staphylococcus* aureus also induced IL‐1α secretion in HaCat cells and human keratinocytes [32]. Here we observed an enrichment of *staphylococcus* genus in the skin upon high humidity exposure. However, we did not find the enrichment of *Staphylococcus* aureus, which could be due to the poor annotation for specific species with 16s sequencing technology. Among the enriched species of *staphylococcus* genus, *S. nepalensis* had the highest disease contribution in random forest analysis and was capable of upregulating IL‐15Rα expression on keratinocytes. In addition, skin re‐colonization of *S. nepalensis* significantly exacerbated psoriasis relapse and increased skin CD8^+^ T_RM_ accumulation not only in mice with psoriasis relapse but also in a humanized animal model with human skin from psoriasis patients, while antibacterial treatment with mupirocin reversed the changes induced by *S. nepalensis* re‐colonization. Therefore, high humidity may aggravate the psoriasis relapse by enriching *S. nepalensis* in the skin of psoriatic mice. Moreover, it was found that *S. nepalensis* reduced the biogenic amines (BAs) in fermented food, which is a risk to human health [52]. However, there is also evidence that *S. nepalensis* is pathogenic. *S. nepalensis* significantly increased neutrophil infiltration and promoted alveolar epithelial cell apoptosis, worsening pulmonary fibrosis [53]. In the present study, we demonstrated that *S. nepalensis* augmented IL‐15Rα expression on keratinocytes and increased the CD8^+^ T_RM_ formation/accumulation, thus exacerbating the recurrence of psoriasis.

Our finding that inhibition of NF‐κB reverses *S. nepalensis*‐induced IL‐15Rα upregulation in keratinocytes aligns with established regulatory mechanisms, wherein NF‐κB is a direct transcriptional activator of the *IL15* gene [33, 54]. This pathway is notably operative in vitiligo skin, as oxidative stress enhances IL‐15/IL‐15Rα expression in keratinocytes in an NF‐κB‐dependent manner [34]. Further, through metabolomic profiling, we identified a cluster of metabolites uniquely enriched in *S. nepalensis*, but not in the commensal *S. epidermidis*, with the arginine and proline metabolic pathways prominently highlighted. Among the candidate metabolites, ADMA (asymmetric dimethylarginine) was functionally validated as a key effector molecule capable of directly inducing NF‐κB phosphorylation in HaCaT cells. ADMA is endogenously produced from methylated arginine residues in proteins and is classically known as a competitive inhibitor of nitric oxide synthase (NOS), thereby modulating vascular tone and endothelial function. Elevated ADMA levels have been reported in several autoimmune diseases, including rheumatoid arthritis [55]and psoriasis [56]. Mechanistically, ADMA can activate the NF‐κB pathway in endothelial cells [55, 57, 58], macrophage [59] and embryonic fibroblasts [60], leading to the production of TNF‐α, IL‐6, and other proinflammatory cytokine. Our findings have extended this paradigm to the skin, demonstrating that ADMA directly activates NF‐κB signaling in keratinocytes. More importantly, we established a direct causal role for it, as intradermal injection of ADMA in vivo recapitulated the psoriasis aggravation induced by *S. nepalensis* colonization and expansion of both IL‐15Rα^+^ keratinocytes and pathogenic CD103^+^CD69^+^ T_RM_ cells. Thus, ADMA serves not only as a biomarker but also as a functional effector metabolite derived from a specific skin bacterium.

While our findings demonstrate a clear causal link between *S. nepalensis* colonization, ADMA production, and exacerbation of psoriasiform inflammation in preclinical models, it is important to acknowledge several limitations. First, the primary evidence presented here derived from murine and humanized mouse models, which, though highly informative, cannot fully recapitulate the complex genetic, immune, and environmental interactions in psoriasis patients. Notably, we did not perform clinical validation in a psoriasis patient cohort, and the direct correlation between cutaneous *S. nepalensis*abundance, ADMA, and disease severity in humans remains to be established. Furthermore, the microbial and metabolic milieu in psoriatic human skin is considerably more diverse and dynamic than in controlled animal settings; therefore, whether ADMA is a dominant driver or just one of several *S. nepalensis*‐derived mediators in psoriasis patients requires further investigation. Future studies should aim to analyze skin microbiota and metabolomic profiles, particularly ADMA and related metabolites. in well‐characterized psoriasis cohorts, correlating these with disease activity, treatment response, and IL‐15Rα/T_RM_ cell biomarkers. Such translational work will be crucial to determine the clinical relevance of *S. nepalensis* and ADMA as potential therapeutic targets for psoriasis recurrence, especially in patients exposed to high‐humidity environment in Southern coastal areas.

## 4 Conclusion

In summary, our study elucidates a high‐humidity‐driven and microbiota‐dependent pathway that exacerbates the relapse of psoriasis. We demonstrate that a high‐humidity environment promotes cutaneous enrichment of *Staphylococcus nepalensis*, a specific bacterium distinct from the commensal S. epidermidis. This dysbiosis triggers a pathogenic cascade wherein *S. nepalensis* and its specific metabolite ADMA directly activate the NF‐κB pathway in keratinocytes. This activation leads to the upregulation of IL‐15Rα on keratinocytes, which orchestrates the expansion and persistence of pathogenic skin‐resident memory T (T_RM_) cells, thereby fueling the chronic inflammation and disease recurrence. Our results have also been confirmed in a humanized mouse model with human skin from psoriasis patients, although it requires direct clinical studies to broaden the potential application for treating human psoriasis in the near future. These findings have provided a novel, mechanistic understanding of how environmental humidity modulates psoriasis recurrence through specific microbial and metabolic mediators, offering potential targets for preventing or treating psoriasis relapse.

## 5 Methods

### 5.1 Mice

BALB/c mice were purchased from Guangdong Medical Laboratory Animal Center (Fushan, Guangdong, China). IL‐15Rα.Loxp/+, IL‐15Rα.Loxp/Loxp and K14‐Cre mice (all in C57BL/6J background) were purchased from Guangzhou Cyagen Company (Guangzhou, China). Homozygous keratinocyte‐specific IL‐15Rα conditional knock‐out mice (IL‐15Rα.Loxp/loxp‐K14‐Cre) were generated by cross‐breeding of IL‐15Rα.Loxp/Loxp and K14‐Cre mice. WT control mice were IL‐15Rα.Loxp/Loxp without cross‐breeding with K14‐Cre mice. Genotyping was performed by PCR screening of tail DNAs. Primers for IL‐15Rα.Loxp allele are F 5’‐TTAGAAGGTAAACATGGCAGTCAG‐3’ and R 5’‐CTTCCAGATGTCTTTACCCTTTGG‐3’. Primers for K14‐Cre allele are F 5’‐CGATGGGAAAGTGTAGCCTGCA‐3’ and R 5’‐TCCAGGTATGCTCAGAAAACGCC‐3’. Further flow cytometric analyses and immunofluorescent staining of skin keratinocytes were conducted to verify IL‐15Rα‐deficiency. All mice were housed in a specific pathogen‐free (SPF) environment, while the animal protocol was approved by the Ethics Committee of Guangdong Provincial Academy of Chinese Medical Sciences (Approval No.: 2021070).

### 5.2 IMQ‐Induced Relapse of Psoriasis‐Like Skin Lesions and Treatment of Mice

To induce the relapse of psoriasiform skin inflammation, the dorsal skin of BALB/c mice was applied with 62.5 mg imiquimod(IMQ)cream for seven consecutive days. Afterward, all mice underwent 14 days’ recover before the second round application of much lower doses of IMQ (20,8 mg) for five consecutive days. To evaluate the effects of high humanity environment on psoriatic skin lesions, mice were kept in climate incubator at 25°C±2°C with a relative humidity of 90% ± 5% for 8 h/day for one week prior to the first round of IMQ treatment until termination of the experiment, while the control mice were housed in a consistent environment at 25°C ± 2°C with ∼60% humidity. To supply *Staphylococcus nepalensis or Staphylococcus epidermidis*, the bacterial suspensions in PBS (10^9^ CFU/mL) were topically applied daily on the dorsal skin starting one week prior to the first round IMQ treatment until one week afterward. To block IL‐15 or IL‐15Rα signaling using IL‐15‐neutralizing or IgG isotype antibody, mice received five intraperitoneal injections of IL‐15‐neutralizing antibody (BE0315, BioXcell, 25 µg/mice), soluble sIL‐15Rα (147‐IR, R&D, 3 µg/mice) or IgG isotype antibody (BE0089, BioXcell, 25 µg/mice) every three days during and after the first round IMQ treatment.

### 5.3 PASI Score Analysis

Skin lesions were evaluated using the Psoriasis Area and Severity Index (PASI) scoring system. The PASI score was assessed based on three indices: skin erythema, scales, and thickness. Each parameter was independently scored on a scale from 0 to 4, where “0” indicates none, “1” slight, “2” moderate, “3” marked, and “4” very marked. The final PASI score is the total sum of the scores for the three indices.

### 5.4 Human Samples and Ethics Approval

All patients (18–60 years‐old) have been diagnosed with recurrent psoriasis. The patients had no medications for at least three weeks when the biopsy was performed. Patient consent was obtained prior to the skin donation. The moderately lesional skin was obtained by the punch biopsy, while the lesional skin from the same patient was used as skin grafts for different recipient mice in each experiment. Peripheral blood was taken from the same patients who donated the skin. The experimental protocols involving human samples were conducted according to the Declaration of Helsinki (1975) Principles and approved by the Ethics Committee of Guangdong Provincial Academy of Chinese Medical Sciences (Approval No.: BF2022‐149‐01).

### 5.5 Psoriatic Skin Grafting in “Humanized” NSG Mice

Skin recipients were six to eight weeks‐old NSG mice (GemPharmatech Co., Nanjing, China). NSG mice were transplanted with lesional skin (8–10 mm^3^) on a right dorsal flank area and secured with a first‐aid bandage. PBMCs were isolated from blood of same psoriatic patients by Ficoll density gradient centrifugation. 5 × 10^6^ PBMCs in saline were intravenously injected to transplanted mice. Each recipient received PBMCs and a skin graft from the same patient. The transplanted mice were subjected to high‐humidity environment as described early. The mice were sacrificed 10 days after transplantation, with human skin grafts subjected to H&E or immunofluorescence staining.

### 5.6 Histopathology, Immunohistochemistry, and Immunofluorescence

Skin tissues were fixed with 4% paraformaldehyde overnight, dehydrated, and embedded into wax blocks. Skin sections (3.5 µm) were dewaxed and stained with hematoxylin‐eosin (H&E). The epidermal thickness and mastoid index were measured using Image‐pro plus 6.0 software, as previously reported [61], under 200× light microscope. For immunohistochemistry or immunofluorescence, skin paraffin sections were fist soaked in antigen repair buffer to repair the antigens using a high‐pressure cooker for 6–10 min. The antigen repair buffer used for anti‐ki67 Ab(ab16667, Abcam, Cambridge, UK), anti‐k19 Ab (MA5‐31977, Invitrogen, USA) or anti‐IL‐15Rα‐AF594 Ab (SC‐374023, Santacruz, USA)staining is citric acid antigen repair buffer (pH 6.0) and for anti‐CD8 (ab217344/ab17147, Abcam, Cambridge, UK) or anti‐CD103 Ab (ab224202, Abcam, Cambridge, UK)it is Tis‐EDTA antigen repair buffer (pH 9.0). For ki67 staining, skin sections were incubated with primary rabbit anti‐ki67 overnight at 4°C and then HRP‐goat anti‐rabbit IgG (KIT‐5005, Maxim, Fuzhou, China) at room temperature for 30 min. The color was developed using DAB substrate kits (DAB‐2031, Maxim, Fuzhou, China). Ki67 positive cells were measured using Image‐Pro Plus 6.0 software.

For dual‐color immunofluorescent staining of IL‐15Rα and K19, skin sections were incubated with anti‐IL‐15Rα‐AF594 and anti‐K19 Abs overnight at 4°C. And then sections were incubated with Alexa Fluor 488‐goat‐anti rabbit‐IgG (ab150081, Abcam, USA), mounted by DAPI Fluoromount‐G (Southern Biotech, Birmingham, UK) and observed via a fluorescence microscope (ECLIPSE Ti2‐E, Nikon, Japan). For dual‐color immunofluorescent staining of CD8 and CD103, skin sections were incubated with anti‐CD8 Abs overnight at 4°C and followed by further incubation with HRP‐ goat‐anti rabbit‐IgG and Tyramide Signal Amplification (TSA)‐combined fluorescent dyes (TSA‐520)using Four‐color multiple fluorescence immunohistochemical staining kits (abs50028, Absin, China). Then after incubation at 37°C with IHC primary antibody eluent buffer(abs994, Absin, China)for 30 min, sections were incubated with anti‐CD103 Abs overnight at 4°Cfollowed by further incubation with Alexa Fluor 594‐goat‐anti rabbit‐IgG (8889S, CST, USA). Finally, sections were mounted by DAPI Fluoromount‐G and observed by a fluorescence microscope.

### 5.7 Quantitative RT‐PCR (qPCR) Arrays

The total skin RNA was extracted using TRIzol reagent (Invitrogen, USA). cDNA was synthesized from 1 µg of total RNA using a reverse transcription kit. Quantitative real‐time PCR (qPCR) was performed on the ABI 7500 PCR System (Applied BioSystems) using SYBR Green Premix Pro Taq HS qPCR Tracking kits (Accurate Biotechnology) for the qPCR arrays of inflammatory cytokines/chemokines and their receptors (wc‐mRNA0266‐m, Wcgene Biotech, China), with primers pre‐embedded in 96‐well plates. GAPDH, B2m, Hprt1, and Act‐β genes were used as internal standard genes, while the 2^−ΔΔCT^ method was utilized to quantitatively analyze the data.

### 5.8 16S DNA Sequencing

To identify the skin microbiota, skin swab samples from the back of the psoriatic mice were collected for total DNA extraction. The V3 and V4 hypervariable regions of prokaryotic 16S rDNA were amplified using the forward primer 5′‐ACTCCTACGGGAGGCAGCA‐3′ and the reverse primer 5′‐GGACTACHVGGGTWTCTAAT‐3′. Sequencing libraries were prepared using Illumina TruSeq Nano DNA LT Library Prep Kits and then subjected to sequencing using the Illumina MiSeq instrument (Illumina Inc., SanDiego, CA). Denoise raw sequencing was read using the DADA2 pipeline in QIIME2 to obtain Amplicon Sequence Variants (ASVs). The taxonomy was assigned to ASVs using the Greengenes database with the RDP Classifier. Analyses of alpha/beta diversity and differential abundance were used to further process data to identify the community composition and abundance of each bacterial species.

### 5.9 *Staphylococcus nepalensis* and *Staphylococcus epidermidis*

*Staphylococcus nepalensis* (S. nepalensis) and ***Staphylococcus epidermidis* (**S. ***epidermidis*)** were purchased from Beijing BioBioWay Biotechnology Co. (https://www.biobw.org/, bio‐090837, bio‐107363, China). S. nepalensis was cultured in nutrient agar (eptone 5.0 g, beef extract 3.0 g, NaCl 5.0 g, agar 15.0 g and MnSO_4_.H_2_O 5 mg in distilled water 1.0 L) at 30°C with shaking. The number of bacteria were calculated by assessing colony‐forming units (CFU) using traditional bacteriology techniques by measuring the optical density at 600 nm.

### 5.10 Untargeted Metabolomics of Bacterial Supernatants

To identify key NF‐κB‐activating metabolites, bacterial supernatants from *S. nepalensis*, *S. epidermidis*, and blank medium (control) were prepared for untargeted metabolomic analysis. A 50 µL aliquot of each sample was mixed with 150 µL of cold extraction solvent (acetonitrile: methanol, 1:4, v/v) containing internal standards, vortexed, and centrifuged. The supernatant was collected for LC‐MS/MS analysis. After extraction with acetonitrile/methanol (1:4), samples were analyzed via LC‐MS/MS (HSS T3 column; Q Exactive HF, ESI±). Differential metabolites between *S. nepalensis* and *S. epidermidis* supernatants versus blank medium were identified using VIP (VIP > 1) and *p*‐value (*p*‐value < 0.05, Student's t test). Venn diagram analysis was used to identify unique metabolites in *S. nepalensis*. Identified metabolites were annotated using KEGG Compound database (http://www.kegg.jp/kegg/compound/), and annotated metabolites were then mapped to KEGG Pathway database (http://www.kegg.jp/kegg/pathway.html).

### 5.11 Keratinocyte Culture

Murine primary keratinocytes were isolated from the skin of WT, conditional IL‐15Rα CKO (IL‐15Rα.Loxp/loxp. K14‐Cre), and WT control sucking mice (IL‐15Rα.Loxp/Loxp). Briefly, the skin was dissected and the underlying fascia and fat were removed. Then tissue was cut into strips of approximately 0.5 cm ×1.5 cm and digested with 5 mL (4 mg/mL) Dispase II solution diluted with KC medium (PriMed‐iCell‐010, iCell, China) at 4°C for 16–18 h. And then, the epidermis was separated by forceps and incubated with 5 mL TrypLE solution at 37°C for 30 min. The pre‐cold KC complete medium (5 mL) was added to terminate the digestion. The collected cells were then cultured in rat tail collagen‐coated 6‐well or 96‐well culture plates using the KC media. After obtaining 70% to 80% confluency, keratinocytes were differentiated with 1.7 mm CaCl2 for 24 h. HaCaT cells (human keratinocyte cell lines) were cultured in DMEM supplemented with 2 mM L‐glutamine and 10% FBS. Either murine primary keratinocytes or HaCaT cell were treated with PBS or cytokine cocktails (IL‐17A 20 ng/mL, TNF‐α 20 ng/mL, IL‐22 10 ng/mL, IFN‐γ 20 ng/mL, Peprotech, China) for 48 h. To evaluate the effects of *S. nepalensis*, keratinocytes were cultured with the supernatants collected in logarithmic growth period of *S. nepalensis* for 24 h. To verify a role of the NF‐κB pathway in *S. nepalensis*‐induced IL‐15Ra expression on keratinocytes, an NF‐κB inhibitor, BAY 11–7082 (1 µm, MCE, USA), was added to the *S. nepalensis* supernatant as an intervention group.

### 5.12 Western Blot Analysis

Total protein was extracted from HaCaT cells using RIPA lysis buffer. After quantification, the proteins were separated by SDS‐PAGE and transferred to PVDF membranes. The membranes were blocked and subsequently incubated at 4°C overnight with the following primary antibodies from Cell Signaling Technology: anti‐p‐NF‐κB p65 (clone 93H1), anti‐NF‐κB p65 (clone D14E12), anti‐p‐IκBα (clone 14D4), anti‐IκBα (clone 2C8), and GAPDH (14C10, CST). All primary antibodies were diluted at 1:1000. After washing, the membranes were incubated with an HRP‐conjugated anti‐rabbit secondary antibody, and the signals were visualized using an ECL detection system.

### 5.13 In Vitro Validation of a Candidate Metabolite (ADMA) in HaCaT Cells

HaCaT cells were seeded in 6‐well plates and treated with ADMA (20, 40, 80 µm) for 24 h. Single cells were prepared for flow cytometric analysis of phosphorylated‐p65 (p‐p65) expression using PE‐conjugated anti‐p‐p65 Ab (MA5‐37165, Invitrogen, UAS). For immunofluorescence (IF), cells were fixed with 4% paraformaldehyde, permeabilized with 0.1% Triton X‐100, blocked with 5% BSA, and incubated with anti‐p‐p65 Ab (1:200, #3033, CST, USA) followed by Alexa Fluor 488‐conjugated secondary Ab. Nuclei were stained with DAPI.

### 5.14 In Vivo Administration of ADMA in Mouse Models of Psoriasis Relapse

To assess the in vivo effect of the candidate metabolite, asymmetric dimethylarginine (ADMA), we utilized the established IMQ‐induced psoriasiform relapse model. Mice received daily intradermal injections of either ADMA (40 mm in PBS, 50 µL per mouse) or vehicle (PBS) on the shaved dorsal skin, starting one week prior to the first round IMQ treatment until one week afterward. The injection volume was distributed across 9–10 points to ensure local delivery. Skin lesions were scored using the PASI scoring system (erythema, scaling, and thickness). Epidermal thickness was measured from H&E‐stained sections. Flow cytometry was used to quantify the frequency of CD103^+^CD69^+^ T_RM_ cells in CD8^+^ T cells, as well as IL‐15Rα^+^ and p‐p65^+^ cells among K19^+^CD45^−^ keratinocytes.

### 5.15 In Vitro Generation of CD8^+^ T_RM_ Cells

Human peripheral blood mononuclear cells (PBMCs, Gaobo Boren Hospital of Beijing, China) or murine splenocytes were cultured in 96‐well plates at a concentration of 2×10^5^ cells/mL with 20 IU/mL rIL‐2 (Peprotech) and IL‐15 (50 ng/mL) (Peprotech) for three days. Then, the medium was refreshed with 20 IU/mL rIL‐2, IL‐15 (50 ng/mL), and TGF‐β1 (50 ng/mL, Peprotech) for an additional 3 days’ culture. To determine the impact of keratinocytes on CD8^+^ T_RM_ cells, keratinocytes were co‐cultured with PBMCs or splenocytes at a ratio of 1:20 with the similar culture condition. CD8^+^CD69^+^CD103^+^ T cells were measured by FACS on day 6.

### 5.16 Flow Cytometry

Splenocytes and lymph node cells were first isolated for flow analysis. To isolate the skin cells, skin was minced and subjected to enzymatic digestion with 200 U/mL type II collagenase (Life Technologies)and 100 µg/mL DNAse (Roche) in HBSS for 4 h. The cells were collected for staining. For surface staining, 1×10^6^ cells were incubated with antibodies at room temperature for 30 min. Abs used included anti‐mouse‐CD45‐percp‐cy5.5, anti‐mouse‐CD45‐PE‐CY7, anti‐mouse‐CD3‐PE‐cy7 (17A2, 100222, Biolegend), anti‐mouse‐CD4‐APC (RM4‐5, 17‐0042‐82, Invitrogen), anti‐mouse‐CD4‐V4509 (RM4‐5, 560468 BD), anti‐mouse‐CD8‐PE‐CY7 (53‐6.7, Invitrogen, 25‐0081‐82), anti‐mouse‐CD8‐FITC (53‐6.7, 553031, BD), anti‐mouse‐CD69‐PE (H1.2F3, 104508, Biolegend), anti‐mouse‐CD103‐PE‐cy7 (2E7, 121426, Biolegend), anti‐mouse‐IL‐15Ra‐APC (DNT15Ra), anti‐human‐IL‐15Ra‐PE (JM7A4, 12‐7159‐42, Invitrogen), anti‐human‐CD8‐FITC (HIT8a, 300916, Biolegend), anti‐CD69‐PE (H1.2F3, 12‐0691‐82, Invitrogen) and anti‐CD103‐APC (2E7, 17‐1031‐82, Invitrogen). To detect K19 expression, cells were first incubated with rabbit anti‐mouse‐K19 primary Ab (SA30‐06, MA5‐31977, Invitrogen) for 30 min. Cells were then incubated with FITC‐conjugated goat anti‐rabbit secondary Ab at for another 30 min. Dead cells were excluded using the Live/dead fixable dead stain kits (Aqua live/dead; Invitrogen). Stained cells were analyzed using NovoCyte Quanteon flow cytometer (Agilent, USA).

### 5.17 Statistical Analysis

Comparisons of means were conducted using one‐way ANOVA or two‐sided *t*‐test. The data were analyzed using GraphPad Prism 8.0 (GraphPad Software, La Jolla, CA, USA) and are presented as Mean ± SD. Statistical significance was defined as a *p*‐value of less than 0.05.

## Ethics Statement

All experiments involving animals and human samples were approved by the Ethics Committee of Guangdong Provincial Academy of Chinese Medical Sciences (Guangzhou, China), as detailed in the Methods section.

## Conflicts of Interest

The authors declare no conflicts of interest.

## Supporting information




**Supporting File**: advs75315‐sup‐0001‐SuppMat.docx.

## Data Availability

All original data sets are available upon request by researchers. The original datasets of DNA sequencing can be found in a public repository through the link below: https://www.jianguoyun.com/p/Dbu2UXYQgbSaDRixiOkFIAA.
