# Supplementary material for: High Humidity Exacerbates Psoriasiform Skin Disease Relapse by Increasing Tissue‐Resident Memory T Cells via Altering Skin Microbiota
Source: Adv Sci (Weinh). 2026 Apr 16;13(38):e04061. doi: 10.1002/advs.202504061 (PMC13335552; doi:10.1002/advs.202504061)
Supplement: Supplementary file 1 — Supporting File: advs75315‐sup‐0001‐SuppMat.docx. [file ADVS-13-e04061-s001.docx]

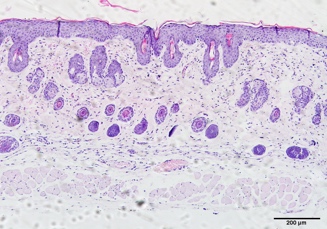

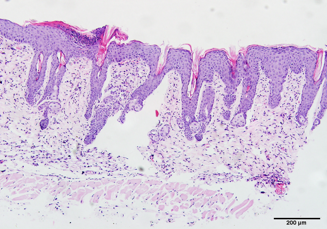

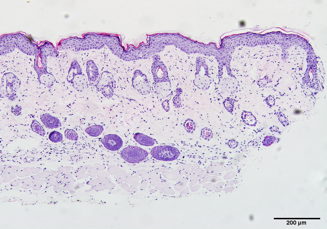

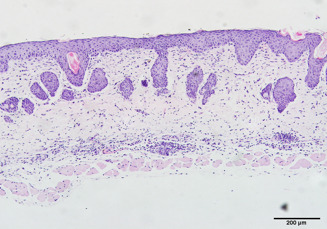


Control

High humidity

+anti-IL-15

+sIL-15Rα

A

B

C

Control

High humidity

+anti-IL-15

+sIL-15Rα

CD103

Control

High humidity

+IL-15Rα

F

CD69

**Day 32**

**Day 34**

D

E

+anti-IL-15


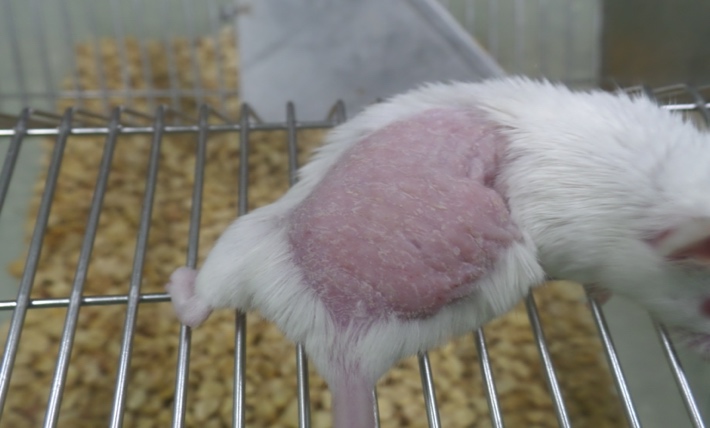

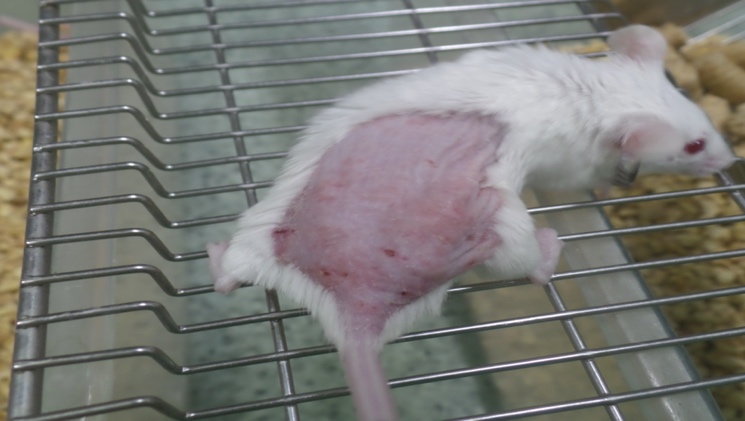

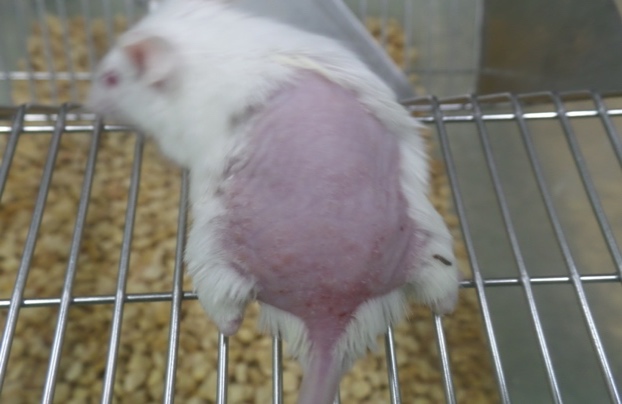

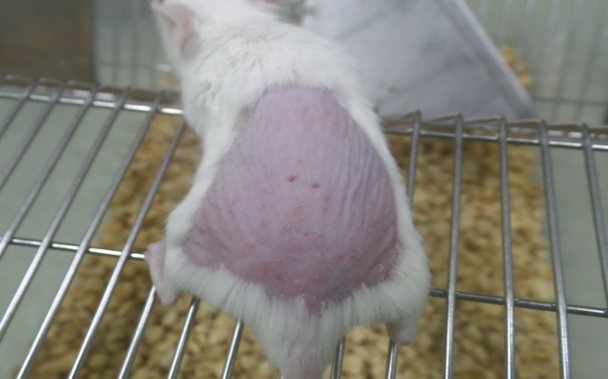

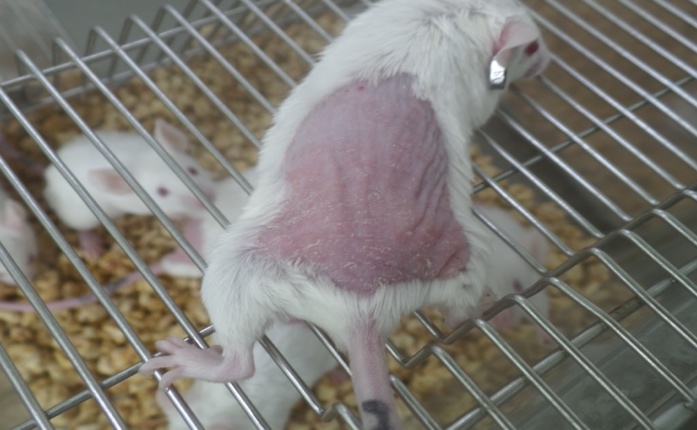

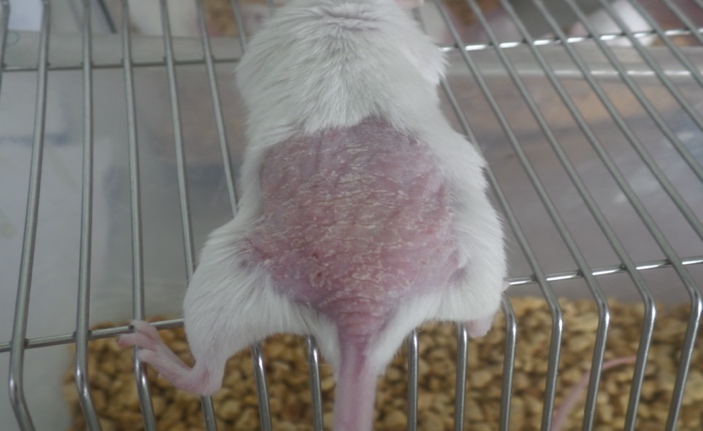

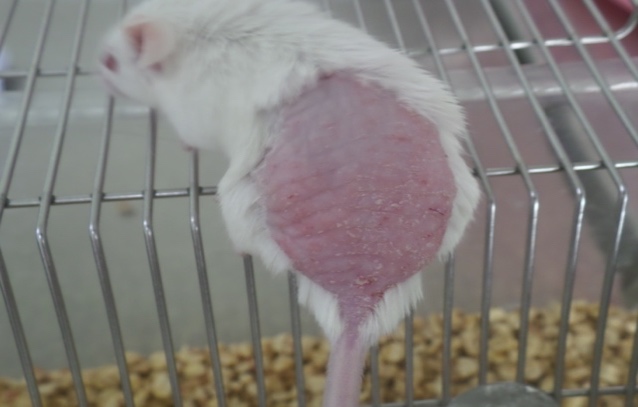

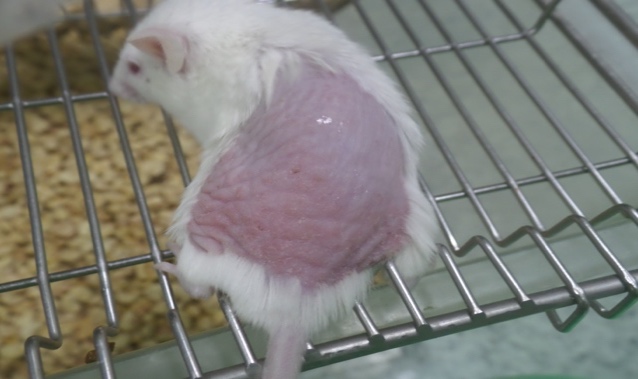


**Figure S1. Blocking IL-15/IL-15Rα axis mostly reverses the murine psoriasis relapse and skin T_RM_ accumulation induced by high humidity.** BALB/c mice were kept in climate incubator at 25±2℃ with a relative humidity of 90% ±5% (High humidity) or 600% ± 5 humidity (Control group) and the psoriasis relapse was induced, as shown in Figure 1A. Mice received five consecutive intraperitoneal injections of IL-15 neutralizing Ab, sIL-15Rα or IgG isotype Ab every three days during and after the first round IMQ treatment. (**A**) Representative images of skin lesions during relapse phase. (**B**) The PASI scores of skin lesions in psoriatic mice during the relapse phase. (**C**) Images of H&E staining of psoriatic skin (200×, Scale bar: 100 μm ). (**D-E**) Measurements of epidermal thickness and papillomatosis indices based on H&E staining. (**F**) Representative dot plots and percentages of CD8^+^ T_RM_ (CD8^+^CD69^+^CD103^+^) cells in CD8^+^ cells in psoriatic skin. Data are presented as mean ±SD from two separate experiments, n=6 mice/group, *p*<*0.05 and **p *<* 0.01.

A

B

**Figure S2. Susceptibility of *S. nepalensis* to an antimicrobial agent Mupirocin**

(**A**) Growth curve of *S. nepalensis* over time*.* (**B**) The two-fold dilution method was used to determine the minimal inhibitory concentration (MIC) of mupirocin on *S. nepalensis*. Shown is an absorbance curve of *S. nepalensis* after 20 hours of culture with different concentrations of mupirocin.

**
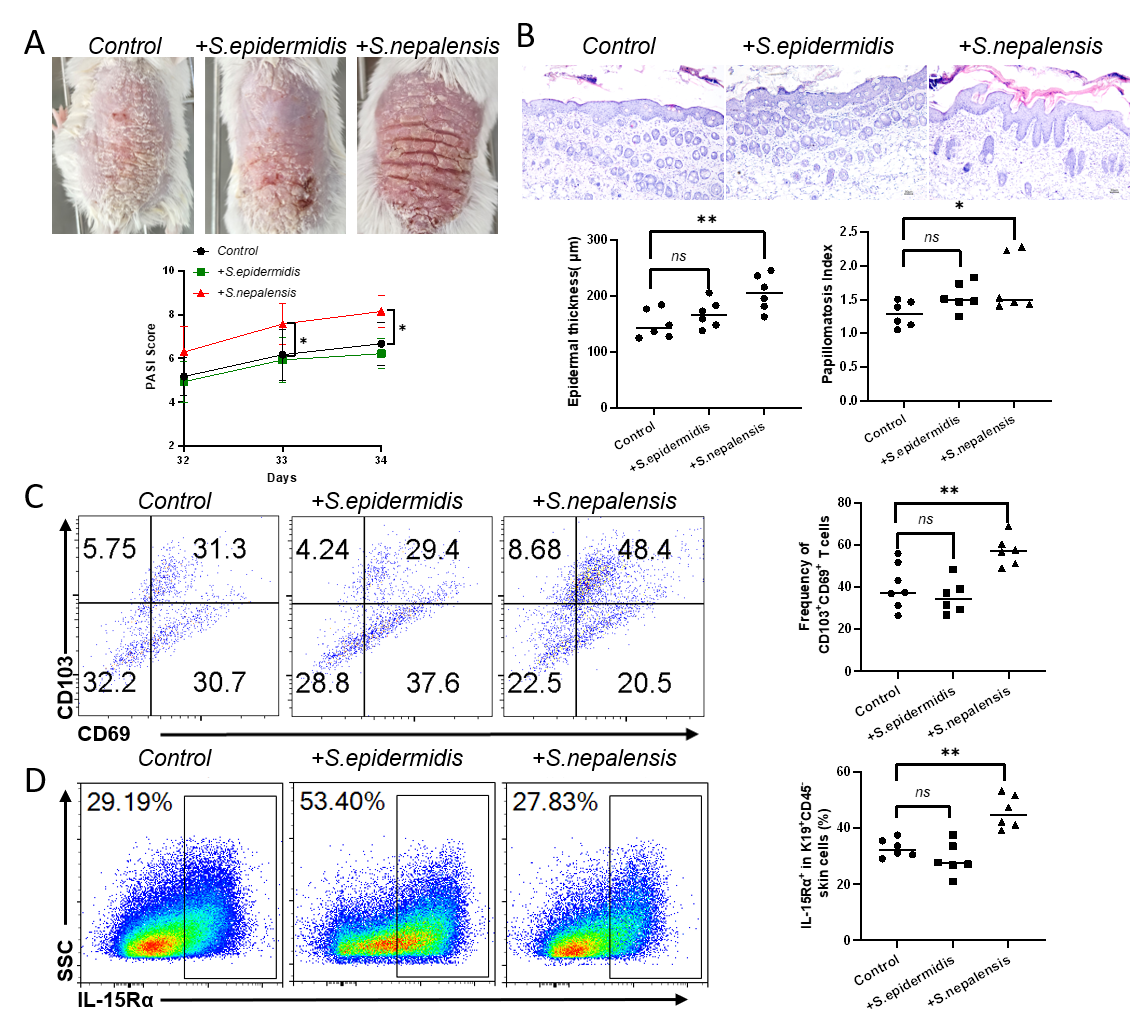
**

**Figure S3.​ As a control, *S. epidermidis* does not exacerbate psoriasis-like inflammation in IMQ-induced mice.** BALB/c mice were kept in climate incubator at 25±2℃ with a relative humidity of 90% ±5% (High humidity) or 60% ± 5 humidity (Control), and the psoriasis relapse was induced, as shown in Figure 1A. Mice inoculated with *S. nepalensis or S. epidermidis* on the dorsal skin using a cotton swab during and after the first round of IMQ treatment as shown in Figure 7. (**A**) Representative images of skin lesions and PASI scores of the lesions during relapse phase. (**B**) Images of H&E staining of psoriatic skin (200×, Scale bar: 100 μm) and measurements of epidermal thickness and papillomatosis indices based on H&E staining. (**C**) Representative dot plots and percentages of CD8^+^ T_RM_ (CD8^+^CD69^+^CD103^+^) cells within CD8^+^ T cells in psoriatic skin. (**D**) Representative dot plots and percentages of IL-15Rα^+^ cells within keratinocytes (CD45⁻K19⁺) in psoriatic skin. Data are presented as mean ±SD from two separate experiments, n=6 mice/group, *p*<*0.05 and **p *<* 0.01.

**Count**

**Hacat Ctrl**

A

**Control**

**S. 1.25%**

**S. 2.5%**

**S. 5.0%**

**Isotype**

B

C

D

**IL-15Rα**

**IL-15Rα**

**Count**

**Control**

**S. 1.25%**

**S. 2.5%**

**S. 5.0%**

**Isotype**

**Pretreated Hacat**

**Figure S4. The supernatant of *S. nepalensis* upregulates the expression of IL-15Rα on keratinocytes.** Control Hacat (**A-B**) or Hacat cells pretreated with cytokine cocktail (**C-D**) for 48h were stimulated with different concentrations of supernatants of *S. nepalensis* for 48h. And then the expression of IL-15Rα on Hacat cells was analyzed via FACS. IL-15Rα expression on Hacat cells treated without or with the supernatant of *S. nepalensis* was shown as mean fluorescence intensity (MFI)*.* Data are presented as mean ± SD from three separate experiments. (*p *<* 0.05 and **p *<* 0.01).

A

C

**S. 5.0%+BAY11-7082**

**Control**

**S. 5.0%**

**Isotype**


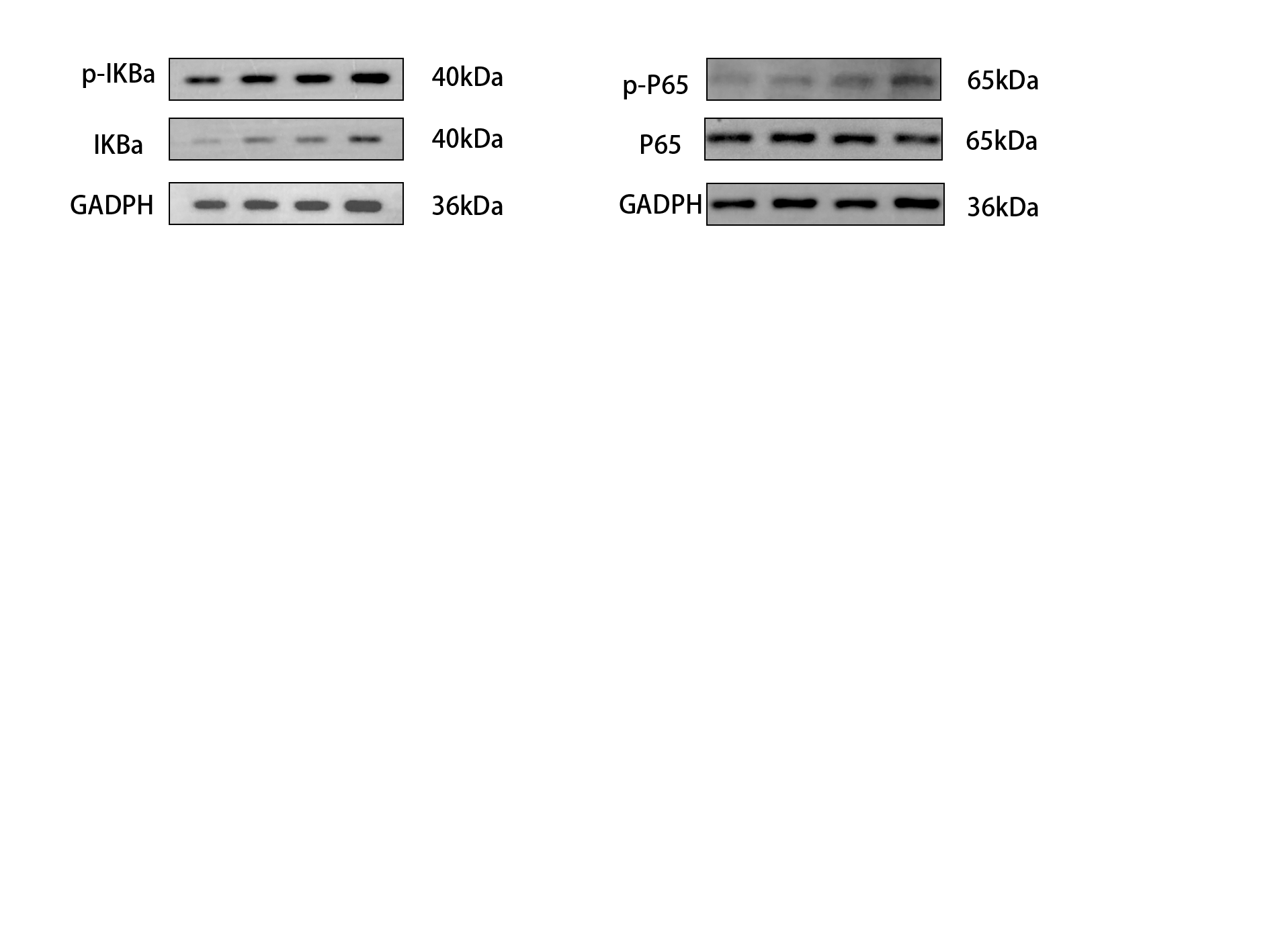


**Control**

**S. 1.25%**

**S. 2.5%**

**S. 5.0%**

**Control**

**S. 1.25%**

**S. 2.5%**

**S. 5.0%**

B

IL-15R𝞪

**Count**

**Figure S5. The supernatant of *S. nepalensis* enhances the NFκB signaling in keratinocytes in vitro.** Hacat cells were stimulated with different concentrations of supernatants of *S. nepalensis* for 24h. And then phosphorated-IKBα (p-IKBα) and p-P65 protein expressions by Hacat cells were measured by Western blot analysis (**A-B**). Moreover, the expression of IL-15Rα on Hacat cells was also analyzed via FACS (**C**). IL-15Rα expression on Hacat cells treated with the supernatant of *S. nepalensis* and an NFκB inhibitor (BAY11-7082) was shown as mean fluorescence intensity (MFI)*.* Data are presented as mean ± SD from three separate experiments. (**p *<* 0.01 and ***p *<* 0.0001).
